# Do nutritional variables improve cardiovascular disease prediction? A comparative machine learning analysis

**DOI:** 10.3389/fnut.2026.1808942

**Published:** 2026-07-08

**Authors:** Kadriye Toprak, Z. Umut Cindiloglu, Nevin Sanlier

**Affiliations:** 1 Department of Nutrition and Dietetics, Faculty of Health Sciences, Ankara Medipol University, Ankara, Türkiye; 2 Intelligent Data Science and Artificial Intelligence Research Center, Universitat Politécnica de Catalunya, Barcelona, Spain

**Keywords:** cardiovascular disease prediction, dietary intake, feature contribution, machine learning, nutritional variables, predictive modeling, random forest

## Abstract

**Introduction:**

Cardiovascular diseases (CVD) remain a leading cause of mortality worldwide, highlighting the need for accurate prediction models. While machine learning (ML) approaches have shown promising results, the contribution of nutritional variables to prediction performance remains unclear. This study evaluates the incremental effect of dietary intake variables by comparing baseline and nutrition-extended feature sets across multiple ML models.

**Methods:**

A cross-sectional dataset of 1,359 adults aged 19–65 years was analyzed, including demographic, anthropometric, clinical, biochemical, and dietary variables collected via 24-h dietary recall. Two feature configurations were constructed: a baseline set excluding dietary variables and a nutrition-extended set including detailed nutritional features. Six ML algorithms—Random Forest (RF), Support Vector Machine (SVM), Logistic Regression (LR), k-Nearest Neighbors (KNN), Decision Tree (DT), and Artificial Neural Network (ANN)–were trained and optimized using cross-validation, with SMOTE applied to the training data to address class imbalance.

**Results:**

The baseline Random Forest model achieved the highest discriminative performance (ROC-AUC = 0.7823). The inclusion of nutritional variables produced limited and model-dependent improvements in selected classifiers (e.g., SVM and ANN) but did not enhance the best-performing baseline model. Across all models, detection of actual cardiovascular disease cases was lower than detection of non-diseased individuals, indicating that further calibration is required before clinical screening use.

**Discussion:**

These findings suggest that nutritional variables provide limited, model-dependent, and incremental contributions to CVD prediction, emphasizing the importance of evaluating feature groups systematically rather than assuming that a larger feature set will improve performance.

## 1 Introduction

Cardiovascular diseases (CVD) remain the leading cause of mortality worldwide, accounting for approximately 17.9 million deaths annually. According to the World Health Organization, nearly 38% of premature deaths (under the age of 70) from non-communicable diseases are attributed to CVD. The etiology of cardiovascular diseases is highly multifactorial, involving complex interactions between genetic predisposition, environmental exposures, and lifestyle-related behaviors (1). Modifiable risk factors such as smoking, physical inactivity, unhealthy dietary patterns, and poor sleep quality, along with clinical parameters including hypertension, dyslipidemia, and hyperglycemia, play a central role in the development and progression of CVD (2). For instance, smoking contributes to endothelial dysfunction and accelerates atherosclerosis through oxidative stress mechanisms (3, 4).

Dietary habits are increasingly recognized as a critical component of cardiovascular health. Nutritional factors, including total energy intake and macro- and micronutrient composition, have been shown to influence cardiometabolic risk (5, 6). Diets characterized by high saturated fat and sodium intake, combined with insufficient dietary fiber and low fruit and vegetable consumption, are strongly associated with increased CVD risk (7, 8). In addition, anthropometric indicators such as body mass index (BMI), waist circumference, and waist-to-hip ratio provide important insights into body fat distribution, which is a key determinant of cardiovascular risk (9, 10). Biochemical markers, including low-density lipoprotein cholesterol (LDL-C), high-density lipoprotein cholesterol (HDL-C), total cholesterol, and triglycerides, further contribute to risk stratification due to their direct involvement in atherosclerotic processes (11–13). Despite the availability of these diverse risk indicators, accurately predicting individual cardiovascular risk remains challenging due to the heterogeneous nature of the disease.

In recent years, artificial intelligence (AI) and machine learning (ML) approaches have gained significant attention in healthcare, particularly in disease risk prediction. ML models are capable of capturing complex, non-linear relationships within high-dimensional datasets and have demonstrated strong performance in cardiovascular risk prediction tasks (14–16). Various studies have reported high predictive accuracy using models such as Random Forest, Support Vector Machines, and neural networks. However, most existing research primarily focuses on clinical, demographic, and biochemical variables, while the role of dietary intake data remains relatively underexplored.

Although some recent studies have begun to incorporate nutritional variables into predictive models, the extent to which these variables contribute to model performance is still unclear (17–21). In particular, there is a lack of systematic investigations comparing baseline models with and without detailed dietary information to evaluate the incremental value of nutritional data.

Therefore, the present study aims to investigate the contribution of nutritional variables to cardiovascular disease prediction by developing and comparing machine learning models using two feature configurations: a baseline dataset comprising demographic, anthropometric, clinical, and biochemical variables, and a nutrition-extended dataset that additionally includes detailed dietary intake information. By evaluating multiple machine learning algorithms under both configurations, this study seeks to provide a comprehensive assessment of whether the inclusion of nutritional variables leads to meaningful improvements in predictive performance.

## 2 Literature review

Machine learning (ML) techniques have been widely applied in recent years for cardiovascular disease (CVD) prediction, demonstrating strong performance across various datasets and modeling approaches. Early studies have primarily focused on optimizing predictive accuracy using clinical, demographic, and biochemical variables. For instance, Sinha et al. (22) proposed the iCardo framework, integrating multiple feature selection techniques such as LASSO, recursive feature elimination (RFE), and chi-square methods to identify optimal predictors, with Support Vector Machine (SVM) achieving the highest accuracy. Similarly, Gupta and Seth (23) compared traditional machine learning and deep learning models using UCI and Framingham datasets, reporting that Random Forest outperformed other models and highlighting the importance of established risk factors such as age and systolic blood pressure. Comparable findings were reported by Mohammad Ali and Ahmed (24), as well as Suleiman et al. (25), who demonstrated that tree-based models, particularly Random Forest, consistently provide robust performance when trained on clinical and anthropometric data.

In addition to model selection, several studies have focused on improving predictive performance through feature engineering, feature selection, and preprocessing techniques. Alfebi and Anasanti (26) emphasized the role of resampling strategies such as SMOTE in addressing class imbalance, while Ghosh et al. (27) proposed hybrid ensemble approaches combining feature selection methods like Relief and LASSO with bagging and boosting techniques, achieving exceptionally high accuracy levels. Other approaches, including clustering-based preprocessing (28) and stacking ensemble models (29), further highlight the importance of data preparation and model combination strategies in improving classification outcomes. However, these studies largely focus on maximizing predictive performance rather than examining the contribution of specific feature groups.

Recent research has also explored more advanced modeling strategies, including hybrid and deep learning-based approaches. Sadr et al. (30) introduced integrated frameworks combining convolutional neural networks (CNN), long short-term memory (LSTM) networks, and traditional ML models, achieving high predictive performance through ensemble learning. While these approaches demonstrate strong accuracy, they often prioritize model complexity over interpretability and provide limited insight into the relative contribution of different data types.

A smaller but growing body of literature has investigated the role of nutritional and lifestyle variables in CVD prediction. Rigdon and Basu (31) reported that incorporating dietary data into machine learning models significantly improved predictive performance, suggesting a synergistic effect between nutritional variables and ML algorithms. Similarly, Morgenstern et al. (17) identified dietary factors such as supplement use, caffeine, and alcohol consumption as important predictors in population-based models. Martin-Morales et al. (18) further compared health-based, nutrition-based, and combined models, concluding that models integrating both clinical and nutritional data achieved superior performance. In addition, Ahiduzzaman and Hasan (32) demonstrated that dietary variables, including micronutrient intake, contribute meaningfully to prediction performance, with interpretable models highlighting their relevance alongside traditional clinical indicators. Dinh et al. (33) also explored the contribution of different data types, showing that the addition of laboratory variables provided only modest improvements in prediction performance.

Despite these advances, the findings regarding the contribution of nutritional variables remain inconsistent across studies. While some research suggests that dietary data significantly enhances predictive performance, other studies indicate only marginal improvements or context-dependent effects. Moreover, most existing studies focus on incorporating nutritional variables directly into predictive models without systematically evaluating their incremental contribution relative to baseline feature sets.

Therefore, there remains a clear gap in the literature regarding the systematic assessment of nutritional variables in CVD prediction. Specifically, there is a lack of studies that explicitly compare baseline models with and without detailed dietary information across multiple machine learning algorithms. Addressing this gap is essential to better understand whether the inclusion of nutritional variables provides meaningful improvements in predictive performance or whether their contribution is dependent on the modeling approach.

## 3 Materials and methods

This section introduces the dataset used in the present study, the data preprocessing procedures, and the machine learning algorithms employed in the analysis.

### 3.1 Study design and participants

This study is based on a secondary data analysis of a community-based cross-sectional field study conducted in several districts of Ankara, Turkey, between August and December 2021. While the original study aimed to comprehensively assess the nutritional and health status of the general population, the present research re-evaluates the dataset to investigate the predictive capacity of multidimensional nutritional variables for cardiovascular disease (CVD) using machine learning approaches.

The study sample consisted of 1,359 adults recruited from the general population using a purposive sampling method. This sampling strategy was employed to ensure the inclusion of participants with sufficiently detailed clinical and nutritional data required for robust predictive modeling. Inclusion criteria were defined as follows: individuals aged between 19 and 65 years at the time of data collection; provision of written informed consent; availability of a complete 24-h dietary recall record; and the presence of biochemical test results obtained within the three months prior to the interview. Exclusion criteria included individuals with cognitive or communication impairments that could compromise the reliability of self-reported data; pregnant or lactating women; participants reporting implausible energy intake values; and individuals with missing or incomplete data on key cardiovascular risk variables.

Ethical approval for the original data collection was obtained from the Ankara Medipol University Non-Interventional Clinical Research Ethics Committee (Date: 27 August 2021, Decision No: 31). The study adhered to the principles outlined in the Declaration of Helsinki. All participants were informed about the purpose of the study, and written informed consent was obtained prior to participation. The present analysis was conducted using anonymized data, and no identifiable personal information was included.

### 3.2 Data collection and measurement

Data were collected through face-to-face interviews using a structured questionnaire developed by the research team. The dataset includes demographic characteristics, lifestyle factors (such as smoking and alcohol consumption), dietary intake, and biochemical parameters.

Anthropometric measurements were obtained by trained researchers following standardized protocols. Body height was measured to the nearest 0.1 cm using a wall-mounted stadiometer, while body weight and body composition parameters—including body fat percentage, total body water, and fat-free mass—were assessed using a Tanita BC-545N multi-frequency bioelectrical impedance analyzer. Waist and hip circumferences were measured using a non-stretch tape measure at the anatomically defined points, in accordance with World Health Organization guidelines. All measurements were conducted with participants wearing light clothing and no shoes.

Dietary intake was assessed using a 24-hour dietary recall method. To improve the accuracy of portion size estimation and reduce recall bias, visual aids such as a standardized food photograph catalog and common household measurement tools were utilized during the interviews. The energy and nutrient composition of the reported foods were calculated using the BEBIS (Nutrition Information System) software.

Biochemical data, including fasting blood glucose and lipid profile parameters (total cholesterol, LDL cholesterol, HDL cholesterol, and triglycerides), were obtained from participants' self-reported laboratory results from the previous three months. The presence of cardiovascular disease was determined based on physician diagnosis as reported by the participants.

All collected data were systematically organized and preprocessed to construct a dataset suitable for machine learning-based analysis.

### 3.3 Dataset description and grouping

The dataset used in the present study was derived from the original field-based assessment and initially consisted of 63 variables collected from 1,359 adult participants. These variables covered a broad range of information, including demographic characteristics, lifestyle factors, anthropometric measurements, biochemical parameters, and detailed dietary intake data. Since the identification (ID) variable had no analytical or predictive relevance, it was removed prior to analysis. Consequently, the final dataset consisted of 62 variables.

To improve interpretability and support the subsequent analytical framework, the variables were organized into two major categories: non-nutritional variables and nutritional variables. The non-nutritional variables included demographic, clinical, lifestyle, anthropometric, and biochemical features, whereas the nutritional variables represented detailed dietary intake measures derived from the 24-hour dietary recall data. This grouping strategy was adopted to facilitate a structured comparison between conventional health-related predictors and the extended feature configuration that additionally incorporated dietary information.

The non-nutritional variables are summarized in Table 1, which presents the variable names, descriptions, domains, data types, and coding structures where applicable. These variables encompass several clinically relevant domains. Demographic characteristics include age, sex, marital status, education level, and occupation. Lifestyle variables include smoking status, number of cigarettes smoked per day, and alcohol consumption. Clinical and disease-related variables include chronic disease status, obesity, diabetes, hypertension, cancer, and physician-diagnosed cardiovascular disease. Anthropometric variables include body height, body weight, body mass index (BMI), waist circumference, hip circumference, waist-to-hip ratio, waist-to-height ratio, neck circumference, body fat percentage, body water percentage, lean mass, abdominal fat, and bone mass. Together, these variables represent the conventional feature space typically used in cardiovascular risk prediction.

**Table 1:** Non-nutritional variables included in the baseline feature set.

Variable	Description	Domain	Type	Coding
Gender	Sex of the participant	Demographic	Categorical	1: Male, 2: Female
Age	Age in years	Demographic	Numerical	Continuous
marital_status	Marital status	Demographic	Categorical	1: Married, 2: Single
Education	Education level	Demographic	Categorical	1: Illiterate, 2: Literate, 3: Primary, 4: Secondary, 5: High School, 6: University+
Occupation	Occupation	Demographic	Categorical	1: Housewife, 2: Officer, 3: Worker, 4: Self-employed, 5: Retired, 6: Paid employee, 7: Unemployed, 8: Student, 9: Other
chronic_disease_status	Presence of any chronic disease	Clinical	Categorical	1: Yes, 2: No
Obesity	Obesity diagnosis status	Clinical	Categorical	1: Yes, 2: No
cardiovascular_disease	Presence of cardiovascular disease	Clinical	Categorical	1: Yes, 2: No
Diabetes	Presence of diabetes	Clinical	Categorical	1: Yes, 2: No
Hypertension	Presence of hypertension	Clinical	Categorical	1: Yes, 2: No
Cancer	Presence of cancer	Clinical	Categorical	1: Yes, 2: No
smoking_status	Smoking status	Lifestyle	Categorical	1: Yes, 2: No, 3: Former
cigarettes_per_day	Number of cigarettes smoked per day	Lifestyle	Numerical	Continuous
alcohol_consumption	Alcohol consumption	Lifestyle	Categorical	1: Yes, 2: No
Height	Height (cm)	Anthropometric	Numerical	Continuous
Weight	Weight (kg)	Anthropometric	Numerical	Continuous
BMI	Body mass index	Anthropometric	Numerical	Continuous
bmi_class	BMI classification	Anthropometric	Categorical	1: Underweight, 2: Normal, 3: Overweight, 4: Obese I, 5: Obese II, 6: Morbid
waist_circumference	Waist circumference (cm)	Anthropometric	Numerical	Continuous
waist_risk	Waist circumference risk level	Anthropometric	Categorical	1: No risk, 2: At risk, 3: High risk
hip_circumference	Hip circumference (cm)	Anthropometric	Numerical	Continuous
waist_hip_ratio	Waist-to-hip ratio	Anthropometric	Numerical	Continuous
whr_risk	Waist-hip ratio risk level	Anthropometric	Categorical	1: No risk, 2: At risk, 3: High risk
waist_height_ratio	Waist-to-height ratio	Anthropometric	Numerical	Continuous
whtr_risk	Waist-height ratio risk level	Anthropometric	Categorical	1: No risk, 2: At risk, 3: High risk
neck_circumference	Neck circumference (cm)	Anthropometric	Numerical	Continuous
neck_risk	Neck circumference risk	Anthropometric	Categorical	1: No risk, 2: At risk
body_fat_percent	Body fat percentage (%)	Anthropometric	Numerical	Continuous
body_water_percent	Body water percentage (%)	Anthropometric	Numerical	Continuous
lean_mass	Lean body mass (kg)	Anthropometric	Numerical	Continuous
abdominal_fat	Abdominal fat level	Anthropometric	Numerical	Continuous
bone_mass	Bone mass	Anthropometric	Numerical	Continuous
fasting_glucose	Fasting blood glucose	Biochemical	Numerical	Continuous
triglycerides	Triglyceride level	Biochemical	Numerical	Continuous
total_cholesterol	Total cholesterol	Biochemical	Numerical	Continuous
LDL	LDL cholesterol	Biochemical	Numerical	Continuous
HDL	HDL cholesterol	Biochemical	Numerical	Continuous
systolic_bp	Systolic blood pressure	Biochemical	Numerical	Continuous
diastolic_bp	Diastolic blood pressure	Biochemical	Numerical	Continuous

The nutritional variables are presented separately in Table 2. These variables include daily energy intake, macronutrient composition, fatty acid subtypes, dietary cholesterol, fiber fractions, fructose, and selected micronutrients such as calcium, magnesium, iron, and caffeine. By distinguishing nutritional variables from the remaining predictors, the present study was able to construct analytically meaningful feature sets and directly evaluate whether the addition of detailed dietary information provides incremental predictive value beyond standard demographic, anthropometric, clinical, and biochemical markers.

**Table 2:** Nutritional variables included in the nutrition-extended feature set.

Variable	Description	Domain	Type	Coding
energy_kcal	Daily energy intake (kcal)	Nutrition	Numerical	Continuous
protein_g	Protein intake (g/day)	Nutrition	Numerical	Continuous
protein_percent	Percentage of energy from protein	Nutrition	Numerical	Continuous
plant_protein_g	Plant-based protein intake (g/day)	Nutrition	Numerical	Continuous
animal_protein_g	Animal-based protein intake (g/day)	Nutrition	Numerical	Continuous
fat_g	Total fat intake (g/day)	Nutrition	Numerical	Continuous
fat_percent	Percentage of energy from fat	Nutrition	Numerical	Continuous
saturated_fat_g	Saturated fat intake (g/day)	Nutrition	Numerical	Continuous
monounsaturated_fat_g	Monounsaturated fat intake (g/day)	Nutrition	Numerical	Continuous
polyunsaturated_fat_g	Polyunsaturated fat intake (g/day)	Nutrition	Numerical	Continuous
dietary_cholesterol_mg	Dietary cholesterol intake (mg/day)	Nutrition	Numerical	Continuous
omega3_g	Omega-3 fatty acid intake (g/day)	Nutrition	Numerical	Continuous
omega6_g	Omega-6 fatty acid intake (g/day)	Nutrition	Numerical	Continuous
carbohydrate_g	Carbohydrate intake (g/day)	Nutrition	Numerical	Continuous
carbohydrate_percent	Percentage of energy from carbohydrates	Nutrition	Numerical	Continuous
fiber_g	Total dietary fiber intake (g/day)	Nutrition	Numerical	Continuous
soluble_fiber_g	Soluble fiber intake (g/day)	Nutrition	Numerical	Continuous
insoluble_fiber_g	Insoluble fiber intake (g/day)	Nutrition	Numerical	Continuous
fructose_g	Fructose intake (g/day)	Nutrition	Numerical	Continuous
calcium_mg	Calcium intake (mg/day)	Nutrition	Numerical	Continuous
magnesium_mg	Magnesium intake (mg/day)	Nutrition	Numerical	Continuous
iron_mg	Iron intake (mg/day)	Nutrition	Numerical	Continuous
caffeine_mg	Caffeine intake (mg/day)	Nutrition	Numerical	Continuous

The descriptive statistics of the numerical variables are reported in Table 3. This table includes the number of available observations (N), mean, standard deviation, minimum value, first quartile (Q1), median, third quartile (Q3), maximum value, missing count, and missing percentage for each continuous variable. These statistics provide an overall characterization of the central tendency, spread, and completeness of the numerical data. As shown in the table, missing data were very limited across the dataset, with missingness observed only in the variable representing the number of cigarettes smoked per day. This pattern is consistent with the data collection process, since participants who did not smoke left this field blank.

**Table 3:** Descriptive statistics of the numerical variables (N = 1,359).

Idx	Variable	N	Mean	Std	Min	Q1	Med.	Q3	Max	Miss. Count	Miss. %
0	age	1,359	47.40	10.60	29.00	38.00	48.00	56.00	65.00	0	0.00
1	cigarettes_per_day	321	12.03	7.61	1.00	6.00	10.00	20.00	40.00	1038	76.38
2	height	1,359	159.78	7.92	138.00	154.00	159.00	164.00	186.00	0	0.00
3	weight	1,359	73.53	13.97	40.40	63.55	72.30	81.40	136.60	0	0.00
4	bmi	1,359	28.87	5.53	16.98	25.02	27.73	32.22	60.16	0	0.00
5	waist_circumference	1,359	95.06	13.76	50.00	85.00	94.00	104.00	147.00	0	0.00
6	hip_circumference	1,359	107.58	10.73	56.00	100.00	106.00	113.00	195.00	0	0.00
7	waist_hip_ratio	1,359	0.88	0.09	0.49	0.83	0.88	0.94	1.39	0	0.00
8	waist_height_ratio	1,359	0.60	0.09	0.34	0.53	0.59	0.66	1.00	0	0.00
9	neck_circumference	1,359	36.08	3.40	28.00	34.00	36.00	38.00	53.00	0	0.00
10	body_fat_percent	1,359	34.84	7.88	9.00	29.60	35.50	40.80	53.50	0	0.00
11	body_water_percent	1,359	45.85	5.56	22.20	42.00	45.40	49.20	92.40	0	0.00
12	lean_mass	1,359	45.04	7.12	30.00	40.10	43.10	47.60	77.80	0	0.00
13	abdominal_fat	1,359	8.44	4.24	1.00	5.00	8.00	11.00	40.40	0	0.00
14	bone_mass	1,359	2.39	0.41	1.10	2.10	2.30	2.50	7.10	0	0.00
15	fasting_glucose	1,359	98.17	27.78	61.00	85.00	92.00	102.00	374.00	0	0.00
16	triglycerides	1,359	131.66	60.35	28.00	92.00	122.00	159.00	549.00	0	0.00
17	total_cholesterol	1,359	189.26	39.81	76.00	159.00	187.00	213.00	400.00	0	0.00
18	ldl	1,359	112.47	31.02	22.00	90.00	110.00	129.00	300.00	0	0.00
19	hdl	1,359	52.43	12.93	13.00	45.00	51.00	59.00	169.00	0	0.00
20	systolic_bp	1,359	120.93	16.30	55.00	110.00	120.00	130.00	199.00	0	0.00
21	diastolic_bp	1,359	74.79	12.40	40.00	68.00	74.00	80.00	121.00	0	0.00
22	energy_kcal	1,359	1480.93	529.03	80.48	1124.78	1414.05	1765.91	5221.68	0	0.00
23	protein_g	1,359	49.43	22.16	3.34	34.42	45.16	59.90	199.64	0	0.00
24	protein_percent	1,359	13.78	3.91	3.00	11.00	13.00	16.00	40.00	0	0.00
25	plant_protein_g	1,359	23.50	11.82	1.25	16.11	22.09	28.38	154.26	0	0.00
26	animal_protein_g	1,359	25.93	17.81	0.00	13.71	21.99	33.60	141.76	0	0.00
27	fat_g	1,359	65.38	27.57	2.22	46.88	61.29	79.50	307.01	0	0.00
28	fat_percent	1,359	39.51	9.11	11.00	34.00	40.00	46.00	64.00	0	0.00
29	saturated_fat_g	1,359	21.96	10.64	1.00	14.57	20.50	26.96	86.43	0	0.00
30	monounsaturated_fat_g	1,359	24.82	11.94	0.74	16.22	22.94	31.36	76.74	0	0.00
31	polyunsaturated_fat_g	1,359	14.29	10.68	0.22	6.82	11.82	18.65	142.50	0	0.00
32	dietary_cholesterol_mg	1,359	206.38	140.90	0.00	89.00	178.40	298.40	911.85	0	0.00
33	omega3_g	1,359	1.07	0.87	0.05	0.69	0.92	1.23	21.15	0	0.00
34	omega6_g	1,359	13.20	10.51	0.17	5.68	10.84	17.40	140.04	0	0.00
35	carbohydrate_g	1,359	169.43	74.47	11.46	120.06	158.48	207.60	724.82	0	0.00
36	carbohydrate_percent	1,359	46.71	9.94	13.00	40.00	46.00	54.00	78.00	0	0.00
37	fiber_g	1,359	17.93	8.47	0.46	12.50	16.94	21.64	104.60	0	0.00
38	soluble_fiber_g	1,359	5.57	2.99	0.18	3.74	5.16	6.86	48.25	0	0.00
39	insoluble_fiber_g	1,359	11.87	5.52	0.28	8.40	11.09	14.43	68.83	0	0.00
40	fructose_g	1,359	13.64	11.06	0.01	5.66	11.02	19.12	103.40	0	0.00
41	calcium_mg	1,359	484.80	251.25	45.50	307.38	445.30	609.54	2506.81	0	0.00
42	magnesium_mg	1,359	203.79	99.16	9.18	141.29	185.63	242.46	1246.20	0	0.00
43	iron_mg	1,359	9.25	4.20	0.37	6.63	8.56	11.05	55.90	0	0.00
44	caffeine_mg	1,359	1.82	30.27	0.00	0.00	0.00	0.00	942.60	0	0.00

The categorical variables were summarized separately in Table 4, which reports the frequencies and percentages of all observed categories. This table provides an overview of the distribution of categorical features across demographic, lifestyle, and clinical domains. The reported percentages were calculated relative to the total sample size, thereby offering a complete view of the dataset composition rather than within-variable proportions only.

**Table 4:** Frequency and percentage distribution of categorical variables.

Variable	Category	Count	Percentage (%)
alcohol_consumption	No	1,316	96.84
alcohol_consumption	Yes	43	3.16
bmi_class	Overweight	537	39.51
bmi_class	Normal	327	24.06
bmi_class	Obese I	311	22.88
bmi_class	Obese II	125	9.20
bmi_class	Morbid	52	3.83
bmi_class	Underweight	7	0.52
cancer	No	1,338	98.45
cancer	Yes	21	1.55
cardiovascular_disease	No	1,132	83.30
cardiovascular_disease	Yes	227	16.70
chronic_disease_status	Yes	941	69.24
chronic_disease_status	No	418	30.76
diabetes	No	1,105	81.31
diabetes	Yes	254	18.69
education	Primary	494	36.35
education	High School	360	26.49
education	University+	311	22.88
education	Secondary	134	9.86
education	Illiterate	33	2.43
education	Literate	27	1.99
gender	Female	1,142	84.03
gender	Male	217	15.97
hypertension	No	963	70.86
hypertension	Yes	396	29.14
marital_status	Married	1,177	86.61
marital_status	Single	182	13.39
neck_risk	At risk	850	62.55
neck_risk	No risk	509	37.45
obesity	No	871	64.09
obesity	Yes	488	35.91
occupation	Housewife	788	57.98
occupation	Retired	257	18.91
occupation	Officer	141	10.38
occupation	Self-employed	66	4.86
occupation	Paid employee	53	3.90
occupation	Worker	35	2.58
occupation	Unemployed	9	0.66
occupation	Other	9	0.66
occupation	Student	1	0.07
smoking_status	No	930	68.43
smoking_status	Yes	321	23.62
smoking_status	Former	108	7.95
waist_risk	High risk	803	59.09
waist_risk	At risk	305	22.44
waist_risk	No risk	251	18.47
whr_risk	At risk	881	64.83
whr_risk	No risk	478	35.17
whtr_risk	High risk	624	45.92
whtr_risk	At risk	570	41.94
whtr_risk	No risk	165	12.14

Because the study aims to predict cardiovascular disease status, special attention was given to the target variable (cardiovascular_disease). Its distribution was examined to assess potential class imbalance before model development. As presented in Table 5, the target variable showed a clearly imbalanced distribution: 1,132 participants (83.3%) belonged to Class 2, whereas 227 participants (16.7%) belonged to Class 1. This imbalance was considered a critical methodological issue for the subsequent modeling phase, as it may influence classifier performance and bias predictions toward the majority class.

**Table 5:** Distribution of the target variable (cardiovascular disease status).

Class	Count	Percentage (%)
2 (No)	1,132	83.3
1 (Yes)	227	16.7

To obtain an initial understanding of the relationships between continuous predictors and the target variable, a correlation analysis was conducted using Pearson correlation coefficients. Although most numerical variables did not strictly follow a normal distribution, Pearson correlation was considered appropriate for exploratory purposes due to the relatively large sample size (*n* = 1, 359) and its usefulness in quantifying the direction and magnitude of linear associations. Overall, most variables exhibited weak to moderate correlations with the target variable. The strongest absolute correlations were observed for abdominal fat (*r* = −0.293), age (*r* = −0.285), and waist circumference (*r* = −0.249). These were followed by triglycerides (*r* = −0.192), neck circumference (*r* = −0.191), total cholesterol (*r* = −0.182), LDL cholesterol (*r* = −0.179), body weight (*r* = −0.177), fasting glucose (*r* = −0.173), and systolic blood pressure (*r* = −0.172). These findings suggest that anthropometric and biochemical variables have relatively stronger linear associations with the target variable than most nutritional variables. In contrast, the majority of dietary predictors showed only very weak correlations. For example, omega-6 intake (*r* = 0.063), omega-3 intake (*r* = −0.061), insoluble fiber (*r* = −0.059), soluble fiber (*r* = −0.057), and total fiber (*r* = −0.055) demonstrated only limited linear relationships, while variables such as energy intake, fat intake, carbohydrate intake, BMI, HDL, and dietary cholesterol were nearly uncorrelated with the outcome. These patterns indicate that no single variable alone strongly explains cardiovascular disease status and support the use of machine learning methods capable of capturing multivariable and potentially non-linear relationships.

Finally, numerical variables were screened for outliers using the interquartile range (IQR) method. The results are presented in Table 6, which summarizes both conventional outliers (1.5 × *IQR*) and extreme outliers (3 × *IQR*). The results indicate that several variables, particularly certain anthropometric, biochemical, and nutritional measures, contained moderate proportions of outlying observations. However, the prevalence of extreme outliers was generally limited across the dataset. Although variables such as fasting glucose, omega-3 intake, and caffeine intake showed relatively higher proportions of extreme values compared to other variables, these observations were retained in the dataset. This decision was made because the values were considered likely to reflect real biological and behavioral variability rather than clear measurement or entry errors. Retaining these observations was therefore deemed more appropriate for preserving the real-world structure of the dataset.

**Table 6:** Outlier analysis of numerical variables based on the IQR method.

Idx	Variable	Q1	Q3	IQR	1.5xIQR Outliers	1.5xIQR %	3xIQR Extreme	3xIQR %
0	age	38.00	56.00	18.00	0	0.00	0	0.00
1	cigarettes_per_day	6.00	20.00	14.00	0	0.00	0	0.00
2	height	154.00	164.00	10.00	28	2.06	0	0.00
3	weight	63.55	81.40	17.85	28	2.06	1	0.07
4	bmi	25.02	32.22	7.21	24	1.77	2	0.15
5	waist_circumference	85.00	104.00	19.00	10	0.74	0	0.00
6	hip_circumference	100.00	113.00	13.00	34	2.50	3	0.22
7	waist_hip_ratio	0.83	0.94	0.12	19	1.40	1	0.07
8	waist_height_ratio	0.53	0.66	0.13	8	0.59	0	0.00
9	neck_circumference	34.00	38.00	4.00	24	1.77	1	0.07
10	body_fat_percent	29.60	40.80	11.20	6	0.44	0	0.00
11	body_water_percent	42.00	49.20	7.20	16	1.18	3	0.22
12	lean_mass	40.10	47.60	7.50	86	6.33	7	0.52
13	abdominal_fat	5.00	11.00	6.00	14	1.03	2	0.15
14	bone_mass	2.10	2.50	0.40	93	6.84	9	0.66
15	fasting_glucose	85.00	102.00	17.00	103	7.58	42	3.09
16	triglycerides	92.00	159.00	67.00	49	3.61	12	0.88
17	total_cholesterol	159.00	213.00	54.00	16	1.18	1	0.07
18	ldl	90.00	129.00	39.00	27	1.99	1	0.07
19	hdl	45.00	59.00	14.00	36	2.65	6	0.44
20	systolic_bp	110.00	130.00	20.00	20	1.47	1	0.07
21	diastolic_bp	68.00	80.00	12.00	61	4.49	11	0.81
22	energy_kcal	1124.78	1765.91	641.14	32	2.35	6	0.44
23	protein_g	34.43	59.90	25.47	44	3.24	6	0.44
24	protein_percent	11.00	16.00	5.00	36	2.65	2	0.15
25	plant_protein_g	16.11	28.38	12.27	34	2.50	11	0.81
26	animal_protein_g	13.71	33.60	19.89	57	4.19	5	0.37
27	fat_g	46.88	79.50	32.62	40	2.94	4	0.29
28	fat_percent	34.00	46.00	12.00	9	0.66	0	0.00
29	saturated_fat_g	14.57	26.95	12.38	44	3.24	4	0.29
30	monounsaturated_fat_g	16.21	31.36	15.14	29	2.13	0	0.00
31	polyunsaturated_fat_g	6.82	18.65	11.84	49	3.61	9	0.66
32	dietary_cholesterol_mg	89.00	298.40	209.40	13	0.96	0	0.00
33	omega3_g	0.69	1.23	0.54	76	5.59	32	2.35
34	omega6_g	5.67	17.41	11.73	51	3.75	8	0.59
35	carbohydrate_g	120.06	207.60	87.55	30	2.21	10	0.74
36	carbohydrate_percent	40.00	54.00	14.00	5	0.37	0	0.00
37	fiber_g	12.51	21.64	9.14	36	2.65	6	0.44
38	soluble_fiber_g	3.73	6.87	3.13	33	2.43	9	0.66
39	insoluble_fiber_g	8.39	14.43	6.03	36	2.65	6	0.44
40	fructose_g	5.66	19.12	13.46	37	2.72	5	0.37
41	calcium_mg	307.38	609.54	302.16	43	3.16	5	0.37
42	magnesium_mg	141.29	242.46	101.17	48	3.53	11	0.81
43	iron_mg	6.63	11.05	4.42	42	3.09	6	0.44
44	caffeine_mg	0.00	0.00	0.00	52	3.83	52	3.83

In summary, the dataset used in this study contains a multidimensional set of predictors representing conventional cardiovascular risk factors as well as detailed nutritional intake data. The structured grouping of variables, together with the descriptive, correlational, and outlier analyses presented in Tables 1–6, provides the methodological basis for the comparative machine learning framework adopted in this study.

### 3.4 Data preprocessing

Prior to model development, a structured preprocessing pipeline was implemented to prepare the dataset for machine learning analysis while preserving the integrity of the original observations and minimizing the risk of information leakage. The preprocessing procedure included missing data handling, outlier inspection, train–test splitting, feature scaling, class imbalance correction, and the consistent application of these steps across both feature configurations used in the study.

#### 3.4.1 Data quality verification and correction

Prior to further preprocessing, a thorough data quality verification was conducted by inspecting the recorded values of all numerical variables. During this inspection, a small number of records were identified as containing values that fell outside physiologically or anatomically plausible ranges. Cross-checking these records against the original questionnaire forms revealed that the inconsistencies originated from data entry errors during the initial digitization of the questionnaires, primarily involving misplaced decimal separators (for example, a BMI of 28.12 mistakenly entered as 2812 (decimal point omitted); a waist-to-hip ratio of 0.87 entered as 87). One record additionally contained an implausible age value (3 years), which was inconsistent with the inclusion criteria.

The following corrections were applied. The single anomalous age value of 3 years was corrected to its accurate value (30 years) following verification against the original questionnaire form. Values of body mass index, waist-to-hip ratio, and waist-to-height ratio affected by decimal-separator errors were recomputed directly from the underlying correctly recorded measurements (height, weight, waist circumference, and hip circumference). Anomalous lean mass values were similarly recomputed using body weight and body fat percentage. After these corrections, all descriptive statistics fell within physiologically plausible ranges, and the full sample of 1,359 adults was retained for analysis.

#### 3.4.2 Handling of missing data

Missing data were minimal in the dataset and were observed only in the variable cigarettes_per_day. This missingness was not considered random, but rather structural in nature. Specifically, participants who reported that they did not smoke typically left the item regarding the number of cigarettes smoked per day blank during the interview process. Therefore, these missing values were interpreted as indicating zero daily cigarette consumption rather than unknown or unrecorded values. On this basis, all missing values in cigarettes_per_day were replaced with 0.

This approach was preferred for both conceptual and analytical reasons. Conceptually, it aligns with the meaning of the variable, since a non-smoker has no daily cigarette consumption. Analytically, it allows the retention of all participants in the dataset without introducing unnecessary missingness handling methods for a variable whose blank values already had an interpretable meaning. As a result, the variable could be used in subsequent modeling without reducing the sample size or distorting the representation of smoking-related behavior.

#### 3.4.3 Outlier inspection and retention

Outlier analysis was performed for all numerical variables using the interquartile range (IQR) method, and both conventional outliers (1.5 × *IQR*) and extreme outliers (3 × *IQR*) were examined. Although several variables exhibited moderate proportions of outlying values, the prevalence of extreme outliers was generally limited across the dataset. In particular, many of the observed extreme values occurred in anthropometric and biochemical variables, such as fasting glucose, triglycerides, abdominal fat, and body composition measures, which may plausibly reflect true biological or clinical variability rather than measurement or recording errors.

For this reason, outliers were not removed from the dataset. Excluding such observations could have resulted in unnecessary information loss, reduced the representativeness of the sample, and potentially removed clinically meaningful high-risk profiles. This consideration is especially important in health-related datasets, where extreme measurements may correspond to individuals with genuinely elevated disease risk. Therefore, instead of deleting these values, the dataset was retained in its original form, and the potential influence of scale differences and extreme magnitudes was later controlled through feature scaling.

#### 3.4.4 Train–test split

After the initial inspection of the dataset, the data were divided into training and test subsets using an 80:20 split. A stratified sampling strategy was used based on the target variable (cardiovascular_disease) in order to preserve the original class distribution in both subsets. This step was performed before any model-related preprocessing operations, such as scaling or oversampling, to prevent information leakage from the test set into the training process.

This procedure is methodologically important because it ensures that the independent test set remains untouched during model development and can therefore provide a realistic estimate of model generalization performance. In classification studies, especially those involving imbalanced medical outcomes, maintaining the original outcome structure in the test set is essential for an unbiased evaluation of predictive accuracy, sensitivity, specificity, and discriminative ability.

#### 3.4.5 Feature scaling

The dataset included numerical variables measured on markedly different scales and in different units, such as age (years), body measurements (cm, kg), biochemical parameters (mg/dL), and nutrient intakes (g, mg, kcal). Because such differences in scale may disproportionately influence model training—particularly in algorithms sensitive to variable magnitude, such as Support Vector Machines, k-Nearest Neighbors, and Artificial Neural Networks—numerical predictors were normalized prior to model fitting.

To achieve this, Min–Max normalization was applied to transform each numerical variable into the [0, 1] range, as shown in Equation 1. This method was selected because it preserves the relative position of observations while bringing all numerical predictors to a common scale.


$$\begin{array}{l} x_{\text{scaled}}=\frac{x-x_{\min}}{x_{\max}-x_{\min}} \end{array}$$ (1)

Importantly, the scaling parameters were estimated only from the training set, and the same transformation was then applied to the test set. This approach was adopted to avoid data leakage and ensure that no information from the independent test set influenced the preprocessing of the training data. In this way, model evaluation remained methodologically valid and reproducible.

#### 3.4.6 Class imbalance handling

As shown in the target distribution analysis, the dataset exhibited a clear class imbalance, with the majority of participants belonging to one outcome category and a substantially smaller proportion belonging to the other. In such cases, machine learning models may become biased toward the majority class, resulting in inflated accuracy but reduced sensitivity for the minority class. This is particularly problematic in disease prediction studies, where the correct identification of at-risk individuals is of primary clinical importance.

To address this issue, the Synthetic Minority Over-sampling Technique (SMOTE) was applied to the training set only. SMOTE generates synthetic samples of the minority class based on the local feature space, thereby balancing the class distribution without simply duplicating existing observations. Applying SMOTE exclusively to the training set is crucial, since performing oversampling on the full dataset or on the test set would artificially inflate model performance and compromise the validity of the evaluation.

Thus, the test set was retained in its original imbalanced form to reflect the natural distribution of the target variable and to provide a realistic assessment of classification performance under real-world conditions.

#### 3.4.7 Consistency across feature configurations

A central objective of the present study was to compare two feature configurations: a baseline dataset containing demographic, lifestyle, anthropometric, biochemical, and clinical variables, and a nutrition-extended dataset that additionally included detailed dietary intake variables. To ensure that any observed differences in predictive performance could be attributed to the inclusion of nutritional variables rather than preprocessing discrepancies, the same preprocessing pipeline was applied to both configurations.

In other words, missing data handling, train–test splitting, scaling, and class balancing were performed according to the same methodological principles for both the baseline and nutrition-extended feature sets. This ensured a fair and internally consistent comparison between the two modeling strategies.

#### 3.4.8 Summary of the preprocessing strategy

Overall, the preprocessing pipeline was designed to balance methodological rigor with preservation of the original clinical and nutritional information contained in the dataset. Missing values were addressed in a conceptually appropriate manner, outliers were inspected but retained to preserve real-world variability, numerical predictors were normalized to a common scale, and class imbalance was corrected in the training data through SMOTE. By performing these operations in a structured sequence and restricting data-driven transformations to the training set, the study aimed to build robust predictive models while minimizing bias and maintaining the validity of the final test evaluation.

### 3.5 Feature configuration and experimental design

A central objective of the present study was to determine whether the inclusion of detailed dietary intake variables provides additional predictive value in cardiovascular disease (CVD) classification beyond that obtained from conventional risk-related variables. To address this objective in a structured and methodologically transparent manner, two distinct feature configurations were constructed and evaluated under the same analytical framework.

#### 3.5.1 Feature configuration

After preprocessing, the full set of predictors was organized into conceptually meaningful domains based on their clinical and functional relevance. These domains included demographic, lifestyle, anthropometric, biochemical, clinical history, and nutritional variables. This grouping strategy was adopted to preserve interpretability and to support a direct evaluation of the incremental contribution of nutritional information.

The baseline feature configuration consisted of variables that are conventionally associated with cardiovascular risk assessment, namely demographic characteristics, lifestyle factors, anthropometric measurements, biochemical parameters, and clinical history variables. In contrast, the nutrition-extended feature configuration included all baseline variables plus the dietary intake variables derived from the 24-hour dietary recall. Accordingly, the two feature sets can be represented as shown in Equation 2 and Equation 3:


$$\begin{array}{c} X_{\text{baseline}}\\ =\left\{X_{\text{demographic}},X_{\text{lifestyle}},X_{\text{anthropometric}},X_{\text{biochemical}},X_{\text{clinical}}\right\} \end{array}$$ (2)

$$\begin{array}{l} X_{\text{nutrition-extended}}=X_{\text{baseline}}\cup X_{\text{nutrition}} \end{array}$$ (3)

where *X*_nutrition_ denotes the set of macro- and micronutrient intake variables obtained from dietary assessment.

This design allowed the present study to go beyond conventional predictive modeling and specifically investigate whether the addition of nutritional variables leads to measurable improvements in classification performance. Rather than assuming that a larger set of predictors automatically yields better results, the study explicitly tested the incremental value of dietary features within a controlled comparative framework.

#### 3.5.2 Experimental design

**Figure 1:**
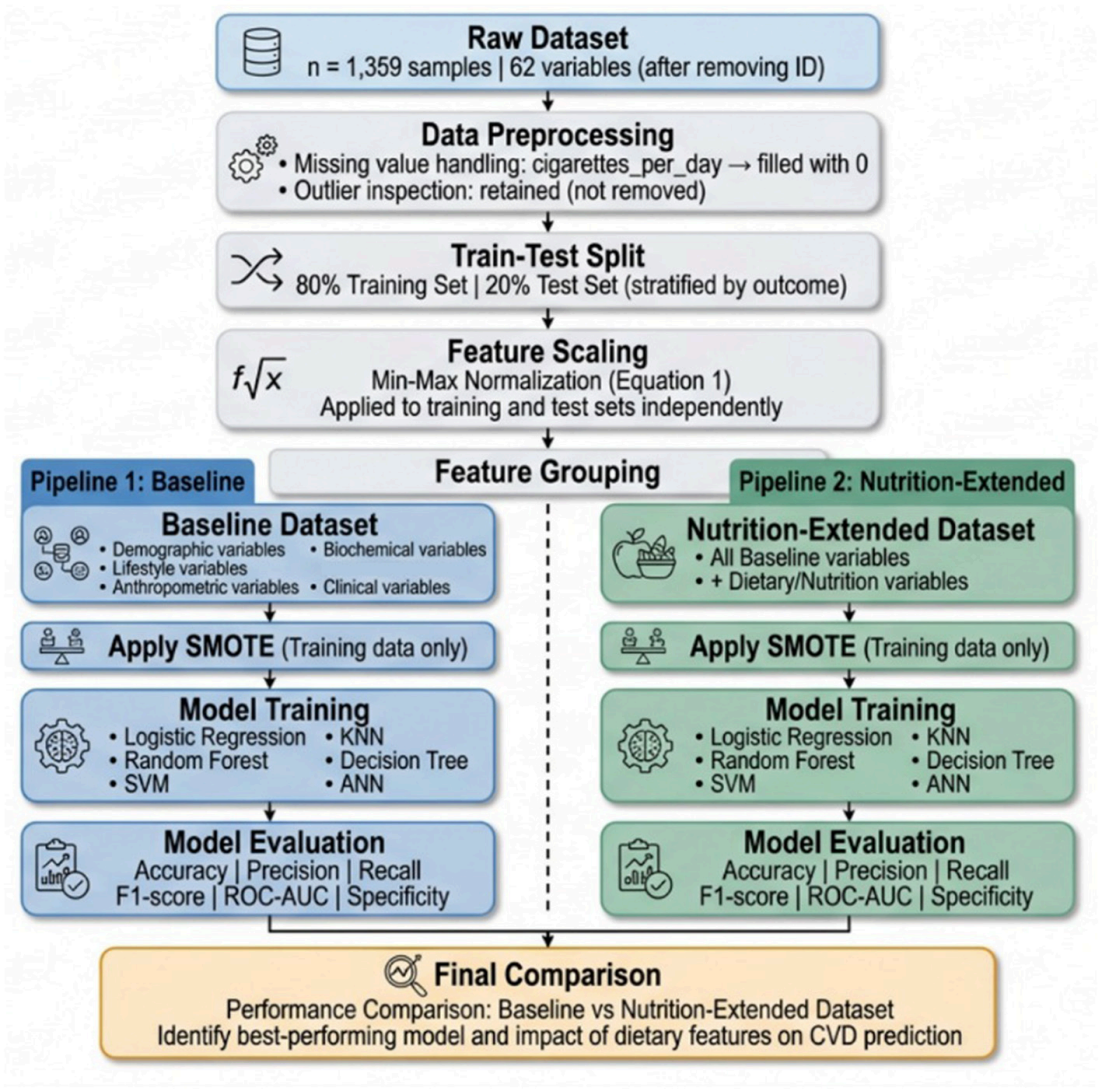
Overall workflow of the comparative machine learning framework used for cardiovascular disease prediction under baseline and nutrition-extended feature configurations. This figure was generated using SciSpace Generate Diagram. The input prompt used to generate it can be found in Supplementary Table 1.

The experimental design of this study was structured to ensure methodological rigor, comparability, and the elimination of potential sources of bias, particularly data leakage. The overall workflow of the analysis is illustrated in Figure 1, which provides a schematic representation of the sequential preprocessing, feature construction, and modeling steps applied in this study.

Following initial preprocessing, the dataset was first divided into training and test subsets using a stratified sampling approach to preserve the original class distribution of the target variable. All subsequent preprocessing operations—including feature scaling and class imbalance handling—were performed in a manner that strictly prevented information leakage from the test set into the training process.

A key component of the experimental design was the construction of two parallel modeling pipelines based on the feature configurations defined earlier: (i) the baseline dataset, and (ii) the nutrition-extended dataset. As illustrated in Figure 1, the workflow branches into two parallel paths after preprocessing and data splitting. Each branch follows an identical sequence of analytical steps, differing only in the inclusion of nutritional variables in the feature set.

To address class imbalance, the Synthetic Minority Over-sampling Technique (SMOTE) was applied independently to the training data within each feature configuration. This step was performed after feature construction, ensuring that the synthetic samples generated reflected the structure of each specific feature space. Applying SMOTE separately to each dataset was methodologically necessary, as performing oversampling prior to feature separation could have introduced dependencies between feature sets and compromised the validity of the comparison. The test set, in contrast, was left unchanged to maintain the natural distribution of the outcome variable and to provide an unbiased evaluation of model performance.

For each feature configuration, multiple machine learning algorithms were trained using the resampled training data. Importantly, the same training and testing partitions were used across both pipelines, ensuring that model comparisons were performed under identical data conditions. This design allowed for a direct and fair evaluation of the impact of nutritional variables on predictive performance.

The overall workflow can be summarized as a sequential process consisting of data preprocessing, stratified train–test splitting, parallel feature construction, independent class balancing within each training set, model training, and final evaluation on a common test set. By maintaining consistency across all analytical steps and isolating the feature configuration as the only varying component, the experimental design enables a valid assessment of the incremental contribution of dietary variables to cardiovascular disease prediction.

As highlighted in Figure 1, this parallel pipeline structure represents the core methodological framework of the study and ensures that any observed differences in model performance can be attributed to the inclusion of nutritional variables rather than to variations in preprocessing or modeling procedures.

#### 3.5.3 Rationale for the comparative design

The comparison between the baseline and nutrition-extended datasets constitutes the main analytical contribution of this study. In the existing literature, nutritional variables are often included as part of a broad predictor pool, but their isolated contribution is rarely examined systematically. As a result, it often remains unclear whether improvements in predictive performance are attributable to dietary information itself, to other clinical variables, or to differences in modeling strategies.

To address this limitation, the present study was designed so that the only structural difference between the two feature configurations was the inclusion of nutritional variables. All other components of the modeling pipeline—including train–test splitting, preprocessing procedures, class imbalance handling, model training, hyperparameter optimization, and evaluation metrics—were kept consistent across both datasets. This design principle can be expressed as Equation 4:


$$\begin{array}{l} \text{ΔPerformance}=f\left(X_{\text{nutrition-extended}}\right)-f\left(X_{\text{baseline}}\right) \end{array}$$ (4)

where *f*(·) represents the predictive performance of a given machine learning model under identical training and testing conditions. Under this formulation, any observed performance difference can be interpreted as the incremental effect associated with the inclusion of nutritional variables, rather than a consequence of differences in preprocessing or model configuration.

This comparative approach provides a more rigorous basis for evaluating the practical relevance of dietary data in CVD prediction. It also allows the analysis of whether the predictive contribution of nutrition is consistent across models or whether it is dependent on the algorithm used.

#### 3.5.4 Analytical purpose of the feature comparison

The analytical purpose of constructing two feature configurations was not merely to determine which model achieved the highest performance, but rather to assess whether dietary intake variables offer additional predictive information beyond conventional cardiovascular risk indicators. In this sense, the present study adopts an incremental modeling perspective, in which nutritional variables are evaluated as an extension of an already informative baseline feature set.

This design is especially relevant from both methodological and practical perspectives. Methodologically, it provides a transparent approach for quantifying the contribution of a specific feature domain. Practically, it addresses an important public health question: whether detailed dietary information meaningfully enhances machine learning-based cardiovascular risk prediction, or whether conventional demographic, anthropometric, and biochemical measures already capture most of the predictive signal.

By structuring the analysis in this way, the study aims to provide a more nuanced understanding of the role of nutritional variables in CVD prediction. Specifically, it allows the identification of whether dietary features improve performance consistently across models, improve performance only for certain algorithms, or fail to contribute additional predictive value when strong conventional predictors are already available.

#### 3.5.5 Summary of the design strategy

In summary, two feature configurations were developed to evaluate the incremental role of nutritional variables in CVD prediction: a baseline dataset including conventional risk-related variables and a nutrition-extended dataset incorporating detailed dietary intake information. Both configurations were analyzed under the same preprocessing, training, and evaluation framework, and class imbalance was handled independently within each training set. This strategy was adopted to ensure a valid and interpretable comparison and to directly address the primary research question of the study.

### 3.6 Machine learning models

To evaluate cardiovascular disease prediction performance under different feature configurations, six supervised machine learning algorithms were employed in this study: Logistic Regression (LR), Decision Tree (DT), Random Forest (RF), Support Vector Machine (SVM), k-Nearest Neighbors (KNN), and Artificial Neural Network (ANN). These models were selected to represent different families of classification methods and to provide a broad comparison across linear, tree-based, distance-based, margin-based, and neural approaches.

The use of multiple algorithms was intentional. Rather than relying on a single classifier, the present study aimed to assess whether the contribution of nutritional variables remains stable across different modeling paradigms or varies depending on the learning mechanism of the algorithm. In this context, the selected models provide a diverse methodological spectrum and allow a more robust interpretation of the predictive role of dietary variables.

Logistic Regression was included as a conventional baseline classifier due to its simplicity, interpretability, and widespread use in clinical prediction research. Because Logistic Regression assumes a linear relationship between predictors and the log-odds of the outcome, it offers a useful reference point against which more flexible machine learning models can be compared. In the present study, its inclusion was particularly relevant for determining whether the nutritional variables contribute predictive information even under a relatively constrained linear framework.

Decision Tree was selected because of its interpretability and its ability to model non-linear decision boundaries through hierarchical splitting rules. Tree-based methods are often appealing in medical applications because their decision logic can be traced and conceptually interpreted. However, single decision trees may be unstable and prone to overfitting, which is why they were included not only as a standalone model but also as a contrast to more robust ensemble approaches.

Random Forest was employed as an ensemble extension of the decision tree framework. By aggregating predictions from multiple trees built on bootstrapped samples and random subsets of predictors, Random Forest reduces variance and generally achieves stronger generalization performance than a single tree. In addition to its strong predictive capability, Random Forest is particularly useful in studies such as the present one because it can model complex interactions and non-linear relationships among predictors without requiring strong distributional assumptions.

Support Vector Machine was included due to its effectiveness in handling high-dimensional classification problems and its ability to capture non-linear decision boundaries through kernel functions. SVM is especially relevant in datasets where the relationship between predictors and outcome may not be adequately represented by a linear separation. Given that dietary, biochemical, and anthropometric variables may jointly interact in complex ways, SVM provided an important alternative perspective within the comparative design.

k-Nearest Neighbors was selected as a distance-based learning method. Unlike parametric classifiers, KNN classifies observations according to the characteristics of nearby instances in the feature space. This makes it highly sensitive to the local geometry of the data and, therefore, particularly informative in relation to preprocessing choices such as feature scaling. Including KNN allowed the present study to examine whether the addition of nutritional variables changes neighborhood structure in a way that improves or reduces classification performance.

Finally, an Artificial Neural Network, implemented as a multilayer perceptron, was used to represent a more flexible non-linear modeling framework. Neural networks are capable of learning complex and potentially non-additive patterns across multiple predictors. Although the dataset used in this study is not large enough to justify deep learning architectures, a shallow ANN offers an appropriate compromise between model flexibility and dataset size. Its inclusion was considered important for evaluating whether nutritional variables provide additional predictive signal when modeled through a non-linear function approximation approach.

Overall, the selected algorithms can be viewed as complementary rather than competing methods. Logistic Regression represents a conventional statistical baseline; Decision Tree and Random Forest capture hierarchical and ensemble tree-based learning; SVM models margin-based non-linear separation; KNN represents local instance-based classification; and ANN captures flexible non-linear patterns through layered transformations. This diversity of classifiers is methodologically important because it enables the present study to investigate not only which model performs best overall, but also whether the effect of adding nutritional variables is consistent across fundamentally different machine learning strategies.

In all cases, the same experimental design was maintained across the baseline and nutrition-extended datasets. Thus, any differences in predictive performance could be interpreted in relation to feature configuration rather than algorithm-specific preprocessing discrepancies. This design choice strengthens the internal validity of the comparison and allows a more direct assessment of the incremental role of dietary variables in cardiovascular disease prediction.

### 3.7 Hyperparameter optimization

The predictive performance of machine learning models is highly influenced by the selection of hyperparameters, which control the structure and learning behavior of the algorithms but are not directly estimated from the training data. In order to obtain models with improved generalization ability and to ensure a fair comparison between algorithms, hyperparameter optimization was performed separately for each classifier within the training data.

In the present study, hyperparameter tuning was conducted using cross-validated search procedures applied only to the training set. This strategy was adopted to avoid information leakage from the independent test set and to ensure that the final test performance reflected true out-of-sample predictive ability. For each model, a predefined hyperparameter search space was constructed based on the methodological characteristics of the corresponding algorithm. Examples include the regularization strength for Logistic Regression, the maximum tree depth and split criteria for Decision Tree and Random Forest, the kernel and penalty parameters for Support Vector Machine, the number of neighbors and distance metric for k-Nearest Neighbors, and the hidden layer size, activation function, and regularization parameter for the Artificial Neural Network.

The optimization procedure aimed to identify the hyperparameter configuration that maximized model discrimination on the training data. Let θ denote the vector of hyperparameters for a given classifier. The optimal hyperparameter configuration can be defined as Equation 5:


$$\begin{array}{l} \theta^{*}=\arg\underset{\theta \in \Theta }{\max}\frac{1}{K}\overset{K}{\underset{k=1}{\sum }}\text{AU}{\text{C}}_{k}\left(\theta \right) \end{array}$$ (5)

where Θ represents the predefined hyperparameter search space, *K* is the number of cross-validation folds, and AUC_*k*_(θ) denotes the ROC-AUC obtained in the *k*-th validation fold using hyperparameter set θ. In this study, *K* = 5 was used through stratified 5-fold cross-validation.

Stratified cross-validation was preferred because the target variable exhibited class imbalance, and preserving approximately the same class proportions in each fold was necessary for obtaining stable and representative validation estimates. Under this procedure, the training set was partitioned into five mutually exclusive folds, each maintaining the original class ratio as closely as possible. At each iteration, four folds were used for model fitting and one fold was reserved for validation. This process was repeated five times, so that every fold served once as a validation set.

The average cross-validated ROC-AUC across the five folds was used as the optimization criterion, because ROC-AUC provides a threshold-independent measure of discriminative performance and is particularly suitable for imbalanced binary classification problems. This is especially relevant in cardiovascular disease prediction, where the goal is not merely to maximize overall accuracy, but to improve the model's ability to distinguish between individuals with and without disease.

Hyperparameter optimization was performed independently for each model and for each feature configuration (baseline and nutrition-extended). This means that the optimal parameters were not assumed to be transferable from one feature set to another. Such separation was methodologically important because the addition of nutritional variables changes the dimensionality and structure of the feature space, which may alter the model's optimal configuration.

The final selected model for each algorithm was then retrained on the full training data using the optimal hyperparameter set θ^*^, and subsequently evaluated on the independent test set. This process can be summarized as Equation 6:


$$\begin{array}{l} {\hat{f}}^{*}=f\left(X_{\text{train}};\theta^{*}\right) \end{array}$$ (6)

where ${\hat{f}}^{*}$ denotes the final trained classifier, *X*_train_ represents the preprocessed and, where applicable, SMOTE-balanced training data, and θ^*^ is the optimal hyperparameter set identified through cross-validation.

It is important to note that hyperparameter optimization was performed only after all preprocessing steps had been completed on the training data and after the feature configurations had been defined. In particular, class balancing using SMOTE was applied separately within each training feature set prior to model fitting, and the test set remained untouched throughout the tuning process. This design ensured that model selection was based exclusively on training information and that the final evaluation on the test set was free from optimistic bias.

Overall, the hyperparameter optimization strategy adopted in this study was designed to improve model robustness, reduce overfitting, and support a fair comparative evaluation across algorithms and feature configurations.

Following the cross-validated hyperparameter search procedure described above, the optimal hyperparameter values obtained for each machine learning model after the training process are summarized in Table 7. These configurations represent the final hyperparameter settings used to train the production models on the full training set and to generate the test-set performance reported in Section 4.

**Table 7:** Optimal hyperparameter configurations identified for each machine learning model under both feature configurations.

Dataset	Model	Best hyperparameters
Baseline	Random Forest	n_estimators = 300; min_samples_split = 2; min_samples_leaf = 1; max_depth = None
Baseline	SVM	kernel = 'rbf'; gamma = 'scale'; C = 100
Baseline	KNN	weights = 'distance'; n_neighbors = 9; metric = 'manhattan'
Baseline	ANN / MLP	solver = 'adam'; learning_rate_init = 0.0005; hidden_layer_sizes = (64, 32); batch_size = 64; alpha = 0.01; activation = 'relu'
Baseline	Logistic Regression	solver = 'lbfgs'; penalty = 'l2'; C = 10
Baseline	Decision Tree	min_samples_split = 10; min_samples_leaf = 4; max_depth = 20
Nutrition-Extended	Random Forest	n_estimators = 300; min_samples_split = 2; min_samples_leaf = 1; max_depth = None
Nutrition-Extended	SVM	kernel = 'rbf'; gamma = 'scale'; C = 100
Nutrition-Extended	KNN	weights = 'distance'; n_neighbors = 7; metric = 'manhattan'
Nutrition-Extended	ANN / MLP	solver = 'adam'; learning_rate_init = 0.01; hidden_layer_sizes = (128,); batch_size = 32; alpha = 0.01; activation = 'relu'
Nutrition-Extended	Logistic Regression	solver = 'lbfgs'; penalty = 'l2'; C = 10
Nutrition-Extended	Decision Tree	min_samples_split = 10; min_samples_leaf = 2; max_depth = None

### 3.8 Model evaluation metrics

The performance of all machine learning models was evaluated on the independent test set using a set of complementary classification metrics. Because the present study addresses a binary cardiovascular disease prediction problem with an imbalanced outcome distribution, no single metric was considered sufficient to capture all relevant aspects of model performance. Therefore, multiple evaluation criteria were used, including accuracy, precision, recall (sensitivity), specificity, F1-score, and the area under the receiver operating characteristic curve (ROC-AUC).

To define these metrics, the confusion matrix was used as the basis of evaluation. In a binary classification setting, model predictions can be summarized in terms of true positives (TP), true negatives (TN), false positives (FP), and false negatives (FN). In the context of this study, these quantities represent correctly and incorrectly classified individuals with and without cardiovascular disease.

#### 3.8.1 Class labeling convention

In this study, the cardiovascular_disease variable was originally coded as 1 (Yes) and 2 (No) in the dataset. For modeling purposes, the labels were recoded such that class 1 corresponds to the absence of cardiovascular disease (the majority class) and class 0 corresponds to the presence of cardiovascular disease (the minority class). This recoding was performed to align the positive class with the majority class in the original data structure and was applied consistently across all models and feature configurations. Accordingly, throughout this study, the reported recall, precision, and F1-score values are computed with respect to class 1 (absence of cardiovascular disease), while specificity refers to the correct classification of class 0 (presence of cardiovascular disease). Readers should interpret recall as the model's ability to correctly identify individuals without cardiovascular disease, and specificity as the model's ability to correctly identify individuals with cardiovascular disease. ROC-AUC, being a threshold- and label-invariant metric, is unaffected by this labeling choice.

#### 3.8.2 Accuracy

Accuracy represents the proportion of all correctly classified observations among the total number of observations as shown in Equation 7:


$$\begin{array}{l} \text{Accuracy}=\frac{TP+TN}{TP+TN+FP+FN} \end{array}$$ (7)

Although accuracy is widely reported in classification studies, it may be misleading in imbalanced datasets because a model can achieve a high accuracy simply by favoring the majority class. For this reason, accuracy was interpreted together with more class-sensitive metrics.

#### 3.8.3 Precision

Precision quantifies the proportion of predicted positive cases that are truly positive as shown in Equation 8:


$$\begin{array}{l} \text{Precision}=\frac{TP}{TP+FP} \end{array}$$ (8)

In the present study, precision reflects the reliability of positive CVD predictions. A higher precision indicates that when the model predicts the presence of cardiovascular disease, it is more likely to be correct.

#### 3.8.4 Recall (sensitivity)

Recall, also referred to as sensitivity, measures the proportion of actual positive class instances that are correctly identified by the model. Under the labeling convention adopted in this study (Section 3.8.1), recall reflects the model's ability to correctly classify individuals without cardiovascular disease (class 1, the majority class). The clinically more relevant detection of cardiovascular disease cases (class 0, the minority class) corresponds to specificity in this study, and the calculation of recall is expressed as shown in Equation 9:


$$\begin{array}{l} \text{Recall}=\frac{TP}{TP+FN} \end{array}$$ (9)

#### 3.8.5 Specificity

Specificity measures the proportion of actual negative class instances that are correctly classified. Under the labeling convention used in this study, specificity reflects the model's ability to correctly identify individuals with cardiovascular disease (class 0, the minority class). Therefore, in the clinical context of this study, specificity is the metric that most directly corresponds to disease detection ability, whereas recall reflects the correct identification of non-diseased individuals as shown in Equation 10:


$$\begin{array}{l} \text{Specificity}=\frac{TN}{TN+FP} \end{array}$$ (10)

#### 3.8.6 F1-Score

The F1-score provides a harmonic balance between precision and recall and is particularly informative when the class distribution is imbalanced as shown in Equation 11:


$$\begin{array}{l} \text{F}1\text{-score}=2\cdot \frac{\text{Precision}\cdot \text{Recall}}{\text{Precision}+\text{Recall}} \end{array}$$ (11)

A higher F1-score indicates that the model maintains a more balanced trade-off between correctly identifying class 1 individuals (non-diseased, under the labeling convention adopted in this study) and limiting false-positive classifications.

#### 3.8.7 Receiver operating characteristic curve and ROC-AUC

In addition to threshold-dependent metrics, the discriminative ability of each model was assessed using the receiver operating characteristic (ROC) curve and its associated area under the curve (ROC-AUC). The ROC curve is constructed by plotting the true positive rate against the false positive rate at different classification thresholds. The true positive rate corresponds to recall, while the false positive rate is defined as shown in Equation 12:


$$\begin{array}{l} \text{False Positive Rate}=\frac{FP}{FP+TN} \end{array}$$ (12)

The ROC-AUC summarizes the overall discriminatory capacity of the model across all possible classification thresholds. Mathematically, it can be interpreted as the probability that the classifier assigns a higher score to a randomly selected positive instance than to a randomly selected negative instance. In integral form, the ROC-AUC can be expressed as shown in Equation 13:


$$\begin{array}{l} \text{ROC}\text{-AUC}=\int_{0}^{1}\text{TPR}\left(\text{FPR}\right)d\left(\text{FPR}\right) \end{array}$$ (13)

where TPR denotes the true positive rate and FPR denotes the false positive rate.

ROC-AUC was used as the primary metric for model comparison in this study for several reasons. First, it is threshold-independent and therefore provides a more general measure of discrimination than accuracy or F1-score. Second, it is less sensitive to class imbalance than raw accuracy. Third, it is especially informative in medical prediction settings, where the classifier's ability to rank individuals according to risk is often as important as its binary classification decisions.

#### 3.8.8 Rationale for metric selection

The choice of evaluation metrics in this study was guided by both statistical and clinical considerations. Since cardiovascular disease prediction is a medically relevant classification problem, emphasis was placed not only on overall predictive performance but also on the model's capacity to identify at-risk individuals. In this context, specificity was considered clinically important because, under the labeling convention adopted in this study, it reflects the extent to which actual cardiovascular disease cases (the minority class) are successfully detected. Recall, in contrast, reflects the correct identification of non-diseased individuals. At the same time, ROC-AUC was prioritized as the principal metric for comparing algorithms and feature configurations because it provides a robust and threshold-independent measure of discrimination.

Accordingly, the interpretation of model performance in this study relied on a combined view of discrimination (ROC-AUC), identification of the majority class (recall), detection of the minority/diseased class (specificity), prediction reliability (precision), and overall balance (F1-score). This multi-metric approach was considered necessary to obtain a comprehensive and clinically meaningful evaluation of model performance.

#### 3.8.9 Final evaluation framework

After hyperparameter optimization had been completed on the training data, the final selected models were evaluated on the independent test set using the metrics defined above. This evaluation procedure was applied identically to both the baseline and nutrition-extended feature configurations. As a result, performance differences between the two configurations could be interpreted as reflecting the contribution of the nutritional variables rather than differences in evaluation methodology.

### 3.9 Software and implementation environment

All data preprocessing, statistical analyses, and machine learning implementations were conducted using the Python programming environment within the Google Colab platform. Google Colab was preferred due to its cloud-based infrastructure, ease of reproducibility, and support for hardware acceleration.

Data manipulation and preprocessing steps were performed using the pandas and NumPy libraries. These tools were used for dataset organization, handling missing values, variable transformations, and preparation of the data for machine learning pipelines.

Machine learning models, hyperparameter optimization, and evaluation procedures were implemented using the scikit-learn library. This framework provided a consistent and efficient interface for training multiple classifiers, performing stratified cross-validation, and computing evaluation metrics across both feature configurations.

To address class imbalance, the Synthetic Minority Over-sampling Technique (SMOTE) was applied using the imbalanced-learn package, which is fully compatible with scikit-learn workflows and allows resampling to be integrated within the training pipeline.

Data visualization tasks, including distribution plots, correlation heatmaps, and graphical summaries of categorical variables, were carried out using Matplotlib and Seaborn, enabling the generation of publication-quality‘figures.

All computations were performed using GPU acceleration available in Google Colab. Although the machine learning models employed in this study are not inherently GPU-dependent, the use of GPU resources improved computational efficiency, particularly during hyperparameter optimization and repeated cross-validation procedures. This contributed to reducing overall training time and enabled more efficient experimentation.

To ensure reproducibility, all stochastic processes—including train–test splitting, cross-validation, and SMOTE resampling—were controlled using fixed random seed values. This approach guarantees that the analysis can be consistently reproduced under the same computational conditions.

Overall, the combination of a cloud-based computational environment, widely used open-source libraries, and controlled experimental settings ensured that the analytical workflow was transparent, reproducible, and computationally efficient.

## 4 Results

### 4.1 Model performance comparison

The comparative predictive performances of all machine learning models under the baseline and nutrition-extended feature configurations are presented in Table 8. Overall, model performance varied across algorithms and evaluation metrics, and the inclusion of nutritional variables produced only limited, model-dependent, and incremental effects, without a uniform pattern of improvement.

**Table 8:** Comparative predictive performance of machine learning models under baseline and nutrition-extended feature configurations.

	CV Best ROC-AUC		Test Accuracy		Test F1-score		Test Precision		Test ROC-AUC		Test Recall		Test Specificity	
Dataset	Baseline	Nutrition-extended	Baseline	Nutrition-extended	Baseline	Nutrition-extended	Baseline	Nutrition-extended	Baseline	Nutrition-extended	Baseline	Nutrition-extended	Baseline	Nutrition-extended
ANN / MLP	0.8807	0.8875	0.6838	0.7059	0.7806	0.8039	0.9273	0.9061	0.7507	0.7583	0.6740	0.7225	0.7333	0.6222
Decision Tree	0.8803	0.8842	0.6949	0.6875	0.8000	0.8028	0.8830	0.8480	0.6967	0.6261	0.7313	0.7621	0.5111	0.3111
KNN	0.9573	0.9617	0.7132	0.7390	0.8160	0.8329	0.8782	0.8939	0.7370	0.7254	0.7621	0.7797	0.4667	0.5333
Logistic Regression	0.9370	0.9379	0.7721	0.7574	0.8584	0.8486	0.8910	0.8852	0.7466	0.7517	0.8282	0.8150	0.4889	0.4667
Random Forest	0.9799	0.9838	0.7978	0.7721	0.8775	0.8622	0.8874	0.8700	0.7823	0.7622	0.8678	0.8546	0.4444	0.3556
SVM	0.9406	0.9466	0.7684	0.7794	0.8571	0.8636	0.8832	0.8920	0.7503	0.7654	0.8326	0.8370	0.4444	0.4889

Under the baseline configuration, the Random Forest model achieved the highest overall discriminative performance, with a test ROC-AUC of 0.7823, which was also accompanied by the highest test accuracy (0.7978) and the highest F1-score (0.8775) among all baseline models. The SVM and ANN/MLP models showed similar discriminative ability, with ROC-AUC values of 0.7503 and 0.7507, respectively, while Logistic Regression also yielded a comparable result (ROC-AUC = 0.7466). In contrast, the Decision Tree model demonstrated the lowest baseline performance, with a ROC-AUC of 0.6967.

With respect to recall, the baseline Random Forest model achieved a high value of 0.8678, indicating strong identification of class 1 (non-diseased) individuals. However, the ANN/MLP model showed the highest test precision (0.9273) under the baseline configuration, despite its lower overall accuracy (0.6838) and lower recall (0.6740). This indicates that the ANN/MLP model was more conservative in assigning class 1 labels, resulting in fewer diseased individuals being misclassified as non-diseased but also a larger number of non-diseased individuals being misclassified as diseased relative to the stronger-performing baseline models.

Under the nutrition-extended configuration, the ranking of models changed slightly. The SVM model achieved the highest discriminative performance, with a test ROC-AUC of 0.7654, followed closely by Random Forest (0.7622) and ANN/MLP (0.7583). Logistic Regression remained relatively stable (ROC-AUC = 0.7517), while KNN and Decision Tree produced lower performance values, with ROC-AUC scores of 0.7254 and 0.6261, respectively.

Regarding classification accuracy, SVM achieved the highest value under the nutrition-extended configuration (0.7794), whereas the nutrition-extended Random Forest model showed a slightly lower accuracy (0.7721) compared with its baseline counterpart. The ANN/MLP model improved from 0.6838 to 0.7059 in test accuracy after the addition of nutritional variables, while its recall increased from 0.6740 to 0.7225. This indicates a modest gain in identifying class 1 (non-diseased) individuals in the neural network setting when dietary variables were included.

Specificity values were generally lower than recall values across both feature configurations and across most models. For instance, the baseline Random Forest model showed a specificity of 0.4444, which decreased to 0.3556 in the nutrition-extended configuration. Similarly, the baseline SVM model showed a specificity of 0.4444, which increased slightly to 0.4889 after the inclusion of nutritional variables. These results indicate that the models were generally more effective at identifying non-diseased individuals (high recall) than at detecting actual disease cases (lower specificity).

Taken together, the results in Table 8 indicate that the inclusion of nutritional variables produced heterogeneous effects across models. Limited and incremental improvements were observed in SVM, ANN/MLP, and, to a lesser extent, Logistic Regression, whereas Random Forest, Decision Tree, and KNN did not show consistent gains under the nutrition-extended configuration. Thus, the contribution of nutritional variables was limited, model-dependent, and incremental, rather than universally beneficial.

#### 4.1.1 Cross-validation stability

To complement the test-set performance metrics reported in Table 8 and to address the statistical uncertainty inherent in cross-validation, the standard deviations of the ROC-AUC values across the five cross-validation folds were also examined. This information provides insight into the stability of model performance during the hyperparameter optimization stage and allows readers to assess not only the central tendency but also the variability of model performance across folds.

Overall, the standard deviations remained small across all models and feature configurations, ranging approximately from 0.006 to 0.025, indicating that the cross-validated performance estimates were stable and that the selected hyperparameter configurations generalized consistently across the training folds. Under the baseline configuration, the lowest fold-to-fold variability was observed for the Random Forest model (mean ROC-AUC = 0.9799, std = 0.0079), followed by the Decision Tree model (mean = 0.8803, std = 0.0081). The SVM (mean = 0.9406, std = 0.0144), KNN (mean = 0.9573, std = 0.0206), ANN/MLP (mean = 0.8807, std = 0.0190), and Logistic Regression (mean = 0.9370, std = 0.0226) models also exhibited acceptably low variability across folds.

A similar pattern was observed under the nutrition-extended configuration, where the Random Forest model again showed the lowest fold-to-fold variability (mean = 0.9838, std = 0.0063), reflecting the well-known stability advantage of ensemble-based methods. The SVM (mean = 0.9466, std = 0.0168), KNN (mean = 0.9617, std = 0.0154), Logistic Regression (mean = 0.9379, std = 0.0221), and Decision Tree (mean = 0.8842, std = 0.0136) models exhibited similarly stable behavior. In contrast, the ANN/MLP model under the nutrition-extended configuration showed the highest fold-to-fold variability (mean = 0.8875, std = 0.0248), which is consistent with the higher sensitivity of neural networks to initialization and training dynamics, particularly in higher-dimensional feature spaces.

Taken together, the cross-validation stability analysis confirms that the reported performance estimates are statistically robust and that the comparative findings across feature configurations are not driven by random fluctuations in the training process. The consistently low standard deviations observed across all models support the reliability of the predictive comparisons reported in Section 4.

### 4.2 Comparative effect of nutritional variables

A direct comparison between the baseline and nutrition-extended models showed that the addition of dietary intake variables did not consistently improve performance across all algorithms. Instead, the observed changes were limited, model-dependent, and incremental.

Among the evaluated models, SVM showed the clearest improvement after the inclusion of nutritional variables. Its test ROC-AUC increased from 0.7503 to 0.7654, and its test accuracy increased from 0.7684 to 0.7794. The ANN/MLP model also exhibited a modest gain, with ROC-AUC increasing from 0.7507 to 0.7583, accuracy from 0.6838 to 0.7059, and recall from 0.6740 to 0.7225. These results indicate that the neural and margin-based models benefited to some extent from the expanded feature space.

Logistic Regression showed relatively stable performance across the two feature configurations. Its ROC-AUC increased only slightly, from 0.7466 to 0.7517, while its accuracy decreased marginally from 0.7721 to 0.7574. This suggests that the nutritional variables did not substantially alter the performance of the linear classifier.

In contrast, Random Forest, which had the strongest overall baseline performance, did not improve after the addition of nutritional variables. Its ROC-AUC declined from 0.7823 to 0.7622, while its accuracy decreased from 0.7978 to 0.7721 and its specificity dropped from 0.4444 to 0.3556. A more pronounced decline was observed in the Decision Tree model, whose ROC-AUC fell from 0.6967 to 0.6261, accompanied by a decrease in both accuracy and specificity. KNN showed mixed behavior, with a modest increase in accuracy (0.7132 to 0.7390) but a decrease in ROC-AUC (0.7370 to 0.7254), indicating that the added nutritional variables altered classification behavior without improving overall discrimination.

Overall, these comparisons show that the addition of nutritional variables did not lead to a systematic increase in predictive performance. Instead, the magnitude and direction of change depended on the model architecture. The strongest baseline model remained Random Forest, whereas the highest nutrition-extended ROC-AUC was achieved by SVM. This indicates that the contribution of nutritional variables is limited, model-dependent, and incremental, with their benefit not being consistent across all classifiers.

### 4.3 ROC curve analysis

**Figure 2:**
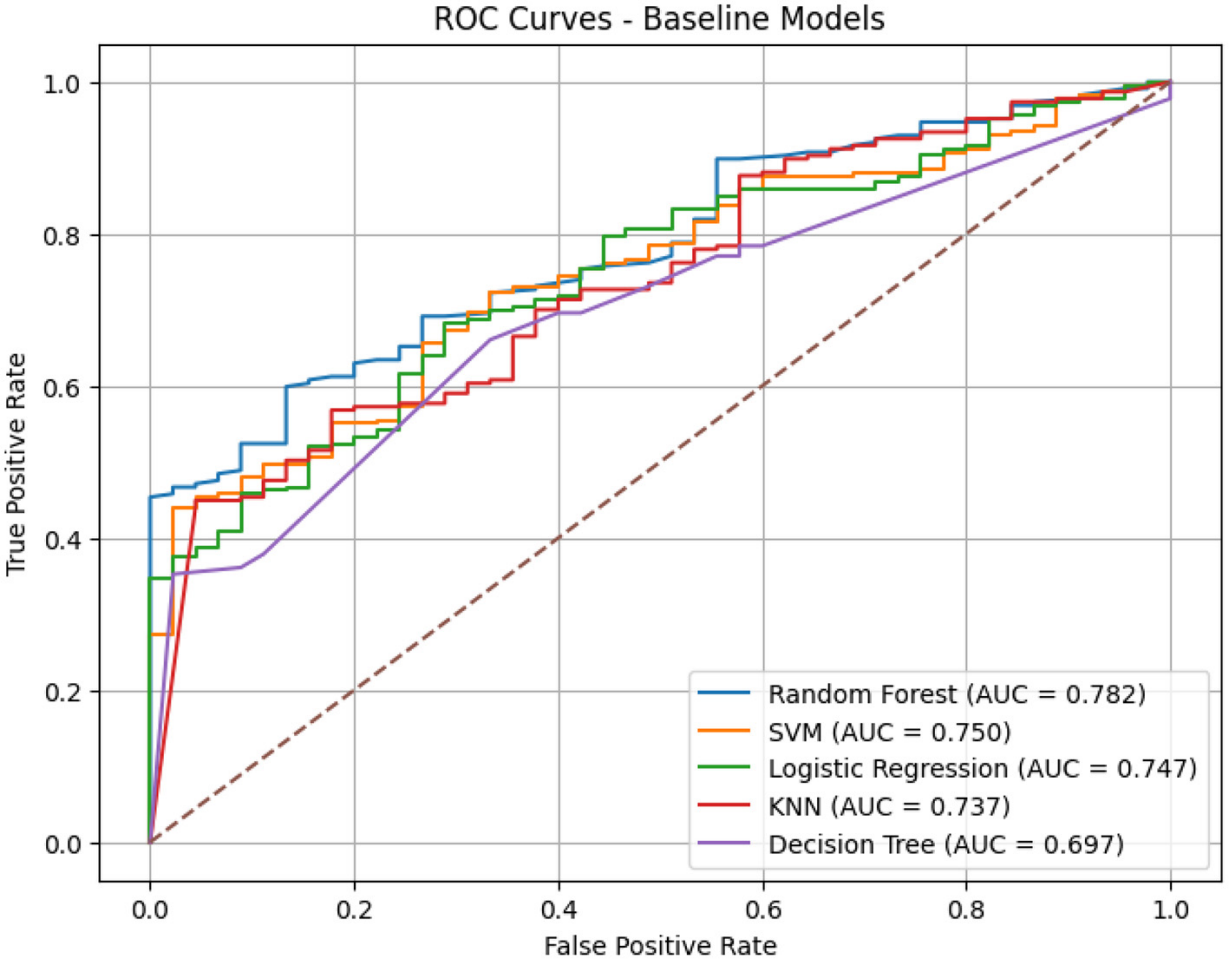
ROC curves of the machine learning models under the baseline feature configuration.

**Figure 3:**
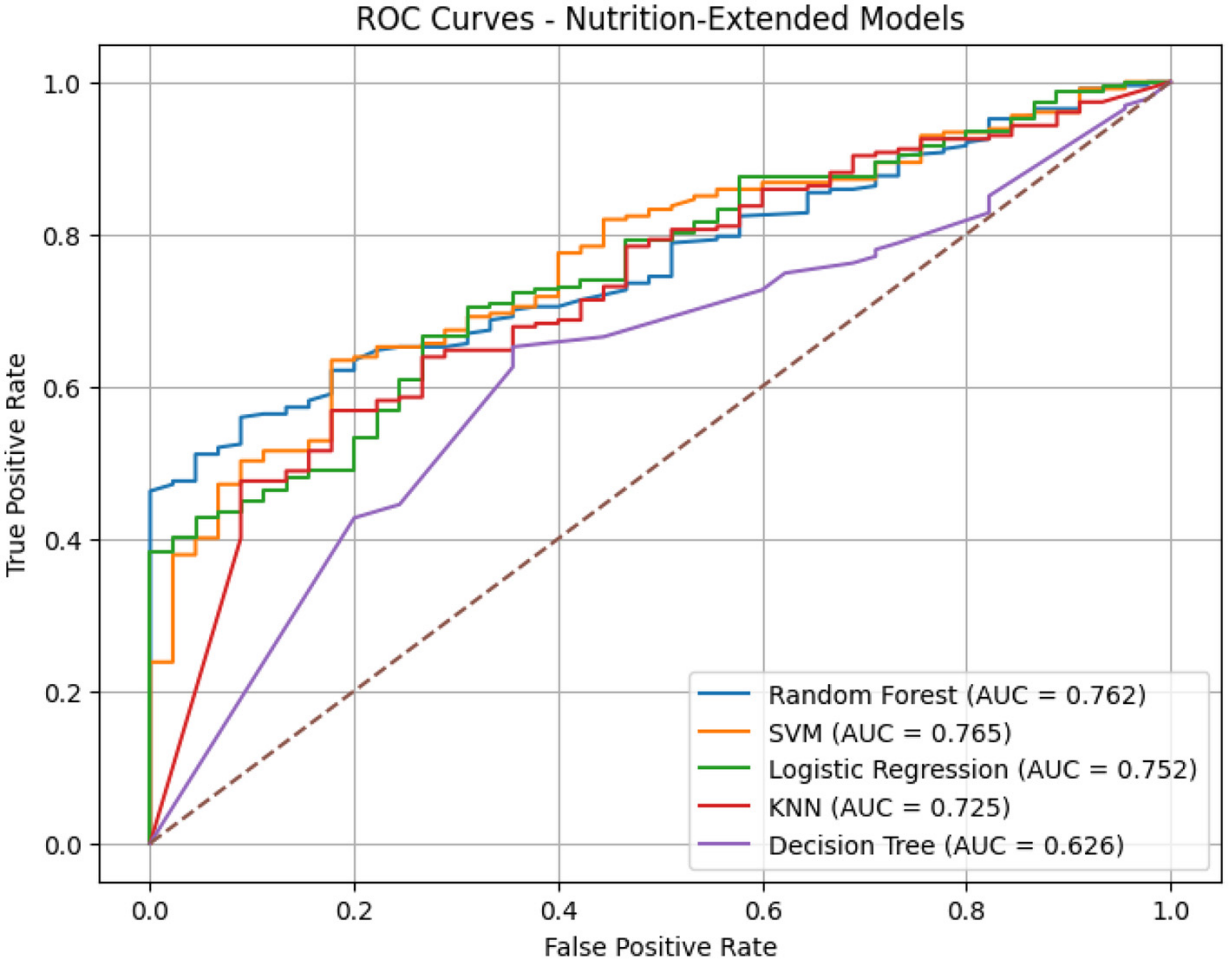
ROC curves of the machine learning models under the nutrition-extended feature configuration.

The receiver operating characteristic curves for the baseline and nutrition-extended models are shown in Figures 2, 3, respectively. These figures provide a threshold-independent visualization of model discrimination by illustrating the relationship between the true positive rate and the false positive rate across all possible classification thresholds.

In the baseline configuration (Figure 2), the Random Forest model showed the most favorable ROC profile, consistent with its highest test ROC-AUC of 0.782. The curves for ANN/MLP, SVM, and Logistic Regression were positioned relatively close to one another, corresponding to their similar ROC-AUC values in the range of approximately 0.747–0.751. The Decision Tree model showed the weakest ROC trajectory, in line with its lower ROC-AUC (0.697).

In the nutrition-extended configuration (Figure 3), the SVM curve was slightly superior to the others, consistent with its highest ROC-AUC of 0.765. The Random Forest curve remained competitive but shifted downward relative to the baseline condition, corresponding to its lower ROC-AUC (0.762) after the addition of nutritional variables. The ANN/MLP model showed a modest upward shift relative to the baseline condition, which is consistent with its numerical improvement in ROC-AUC (0.758). By contrast, the Decision Tree model displayed a clear deterioration in ROC behavior, matching its marked decline in ROC-AUC (0.626).

The ROC curves therefore confirm the findings reported in Table 8. While certain models, particularly SVM and ANN/MLP, showed modest improvement with the nutrition-extended dataset, the strongest baseline model (Random Forest) did not improve under the extended feature configuration. Thus, the visual discrimination patterns reinforce the model-dependent nature of the contribution of nutritional variables.

**Figure 4:**
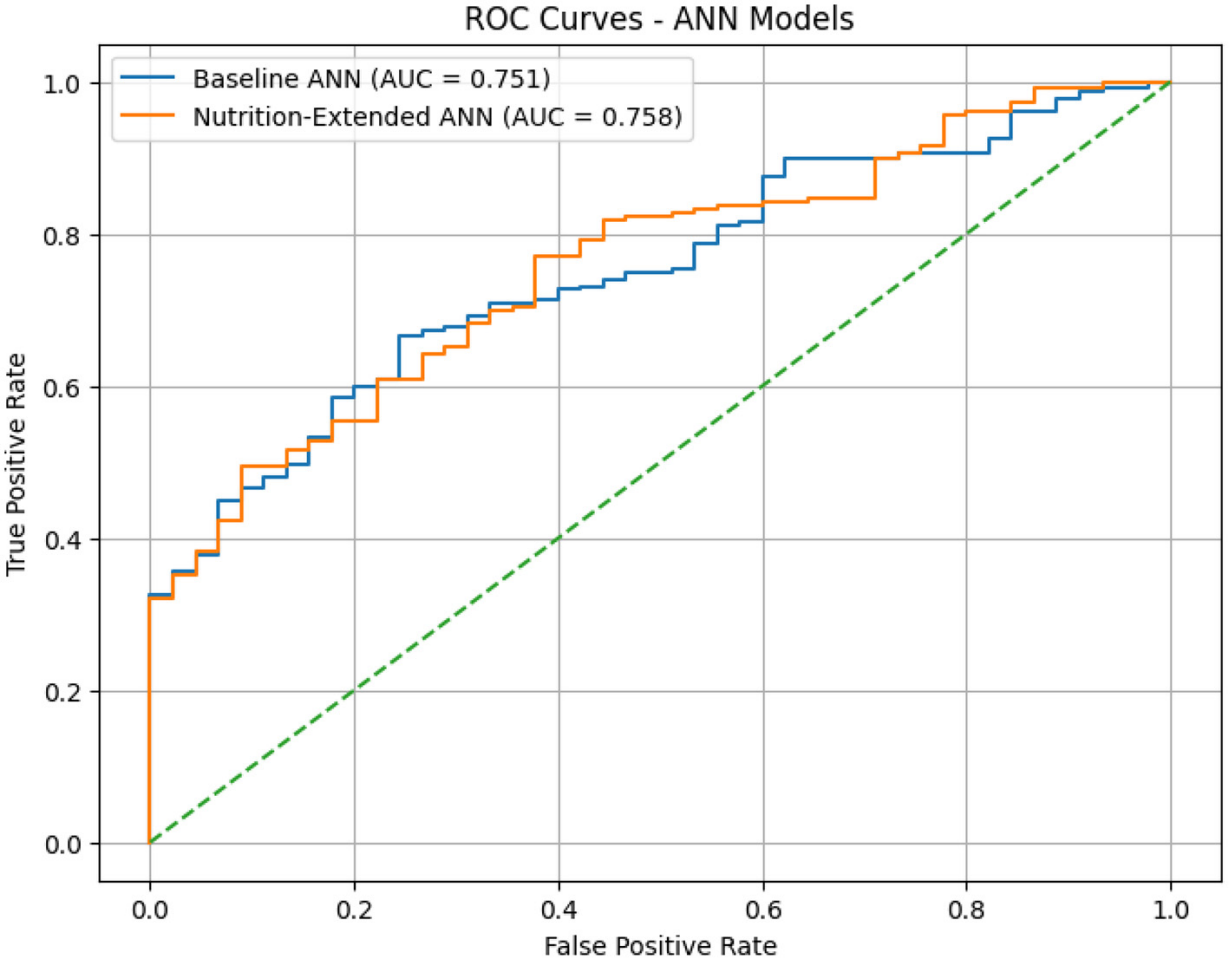
Comparative ROC curves of the baseline and nutrition-extended ANN/MLP models.

Because the ANN/MLP model showed particularly notable changes in precision and recall, its ROC curves are displayed separately in Figure 4. As shown in that figure, the nutrition-extended ANN/MLP model achieved a slightly higher AUC than the baseline ANN/MLP model, confirming a modest improvement in discrimination after the inclusion of nutritional variables.

### 4.4 Confusion matrix analysis

**Figure 5:**
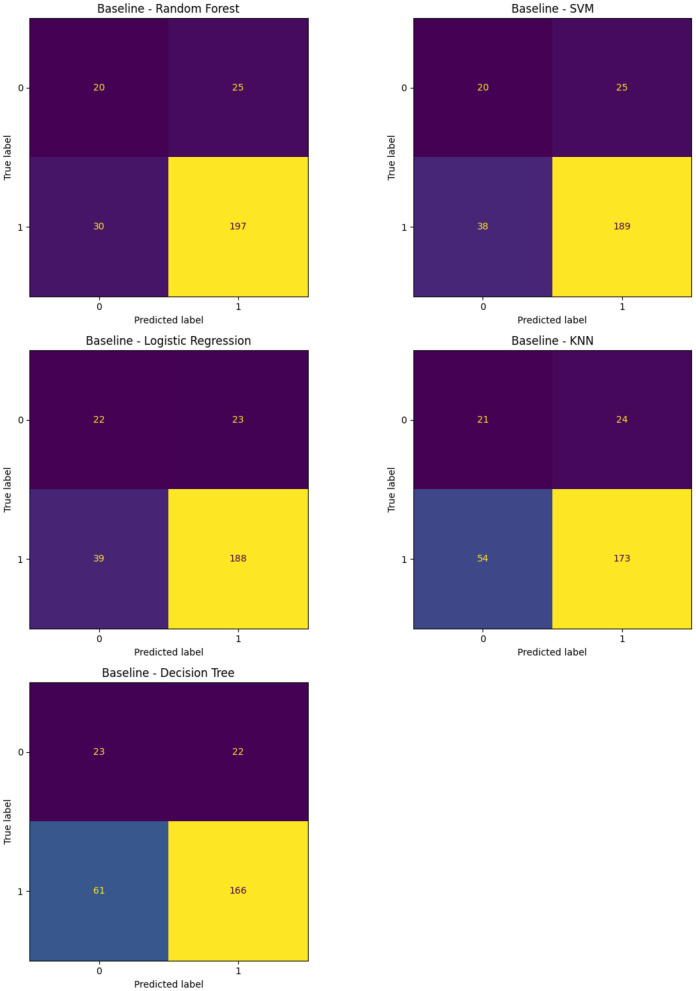
Confusion matrices of the machine learning models under the baseline feature configuration.

**Figure 6:**
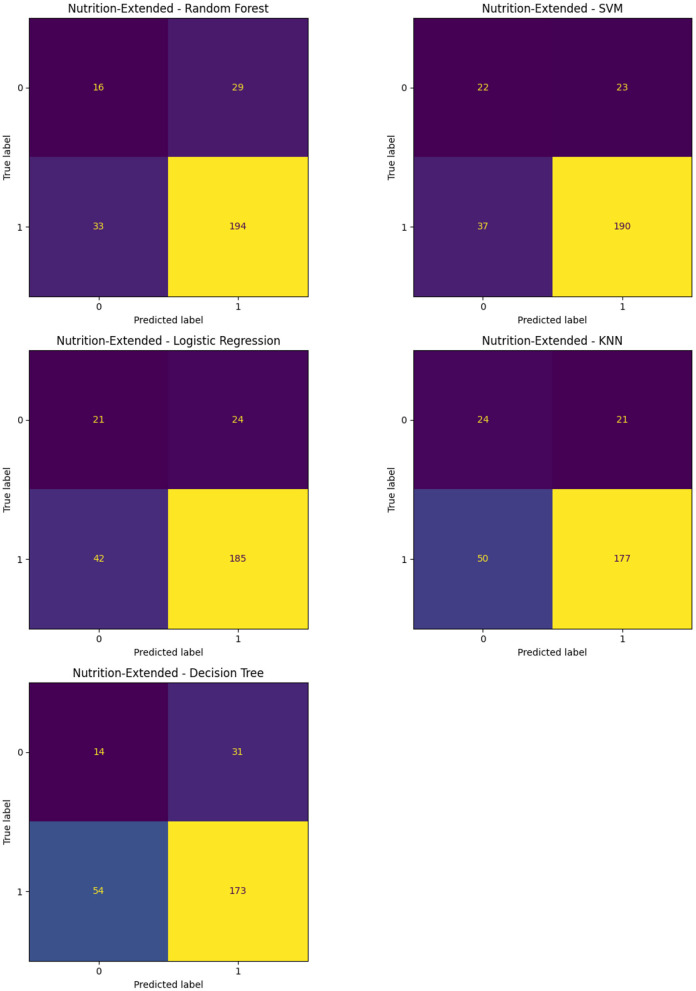
Confusion matrices of the machine learning models under the nutrition-extended feature configuration.

The confusion matrices for the baseline and nutrition-extended models are presented in Figures 5, 6, respectively. These matrices provide a detailed view of classification behavior by showing the distributions of true negatives, false positives, false negatives, and true positives for each model.

In the baseline configuration (Figure 5), the Random Forest model correctly identified 197 non-diseased individuals (class 1) while generating 25 false positives and 30 false negatives, reflecting a relatively balanced classification profile. The SVM model correctly classified 189 non-diseased individuals, but produced 38 false negatives, indicating somewhat lower sensitivity than Random Forest. Logistic Regression showed a similar pattern, with 188 correctly classified non-diseased individuals and 39 misclassified ones. The KNN and Decision Tree models showed larger numbers of false negatives (54 and 61, respectively), corresponding to their lower recall and weaker overall performance.

In the nutrition-extended configuration (Figure 6), the Random Forest model continued to classify a high number of non-diseased individuals correctly (194 correct class 1 predictions), but the number of false positives increased to 29, consistent with the decline in specificity reported in Table 8. The SVM model showed 190 correct class 1 predictions and 37 misclassified class 1 instances, indicating a slightly improved balance relative to its baseline version. Logistic Regression correctly classified 185 non-diseased individuals, while KNN and Decision Tree continued to show relatively high numbers of misclassified class 1 instances (50 and 54, respectively).

**Figure 7:**
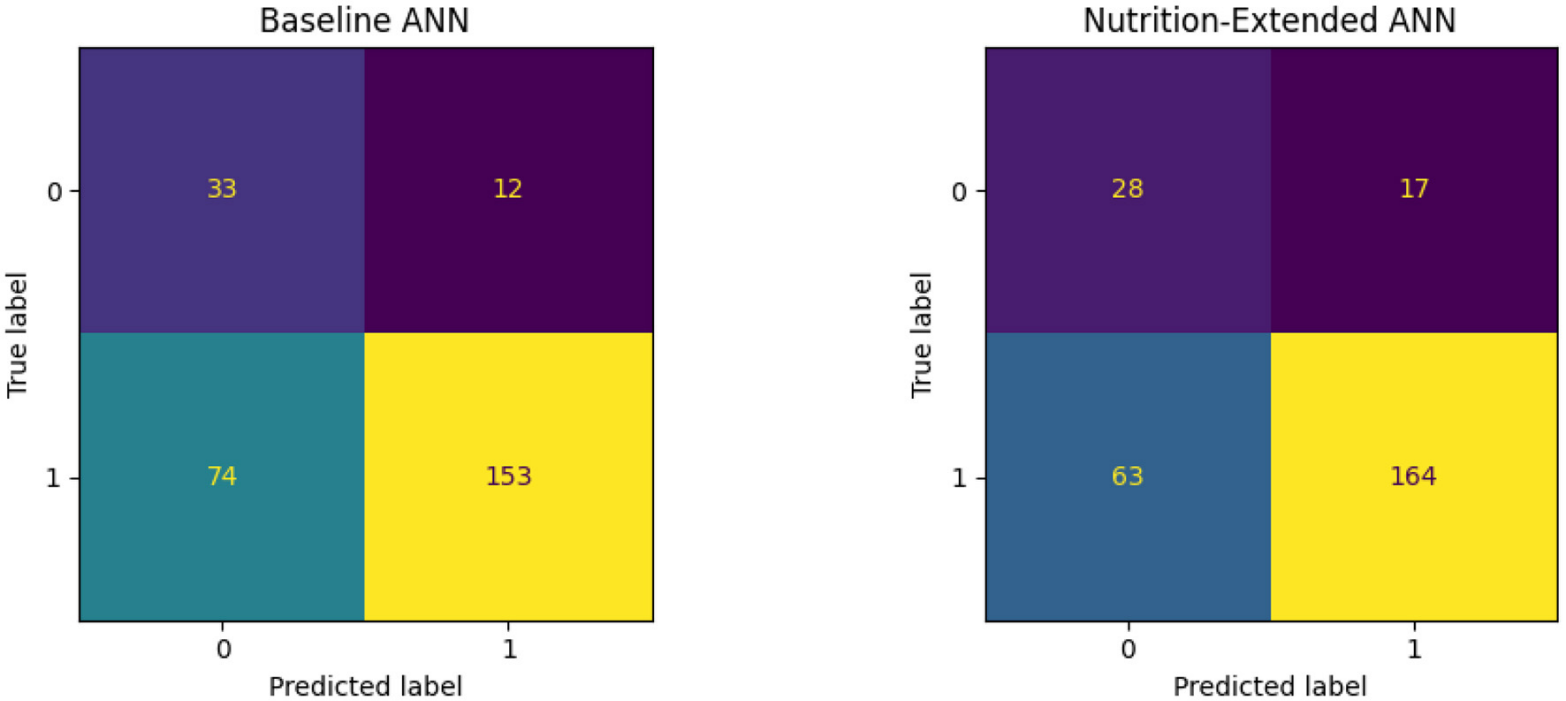
Comparative confusion matrices of the baseline and nutrition-extended ANN/MLP models.

The ANN/MLP confusion matrices are shown separately in Figure 7 because this model demonstrated a distinctive classification pattern. In the baseline ANN/MLP model, the number of true negatives was 33, false positives 12, false negatives 74, and 153 correct class 1 predictions. In the nutrition-extended ANN/MLP model, true negatives decreased to 28, false positives increased to 17, false negatives decreased to 63, and correct class 1 predictions increased to 164. This pattern indicates that the addition of nutritional variables improved the model's identification of class 1 (non-diseased) individuals but at the cost of an increase in diseased individuals misclassified as non-diseased. This is fully consistent with the numerical results, where recall improved while specificity declined.

Overall, the confusion matrix analysis demonstrates that the inclusion of nutritional variables tended to alter the balance between sensitivity and specificity differently across algorithms. In some models, especially ANN/MLP and SVM, the addition of nutritional variables was associated with improved identification of non-diseased individuals. However, this was frequently accompanied by an increase in diseased individuals misclassified as non-diseased or a decline in specificity. These findings further support the conclusion that the predictive contribution of nutritional variables is limited, model-dependent, and incremental, and not uniform across classifiers.

### 4.5 Summary of comparative predictive findings

Taken together, the results presented in Table 8 and Figures 4–7 indicate that the baseline Random Forest model achieved the strongest overall predictive performance in the study, particularly in terms of ROC-AUC, accuracy, and F1-score. Although nutritional variables produced limited and incremental gains in selected models—most notably SVM and ANN/MLP—they did not lead to a consistent improvement across the full set of classifiers. Moreover, the strongest baseline model did not benefit from the addition of nutritional variables. These findings suggest that the inclusion of dietary intake variables influences model behavior differently depending on the underlying learning mechanism, and that their predictive contribution is best characterized as limited, model-dependent, and incremental, rather than universally advantageous.

### 4.6 Feature importance analysis

**Figure 8:**
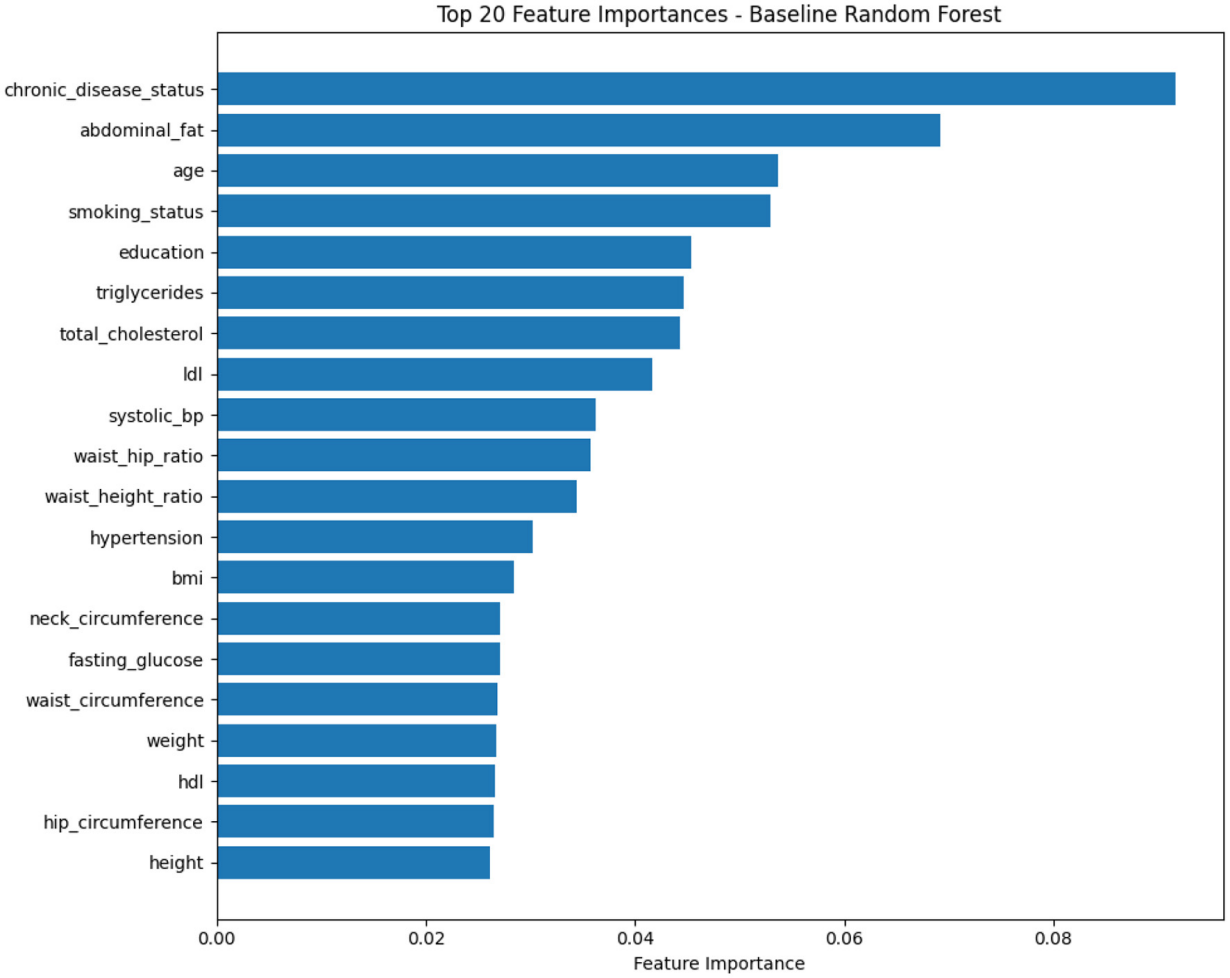
Feature importance plot for the baseline Random Forest model.

**Figure 9:**
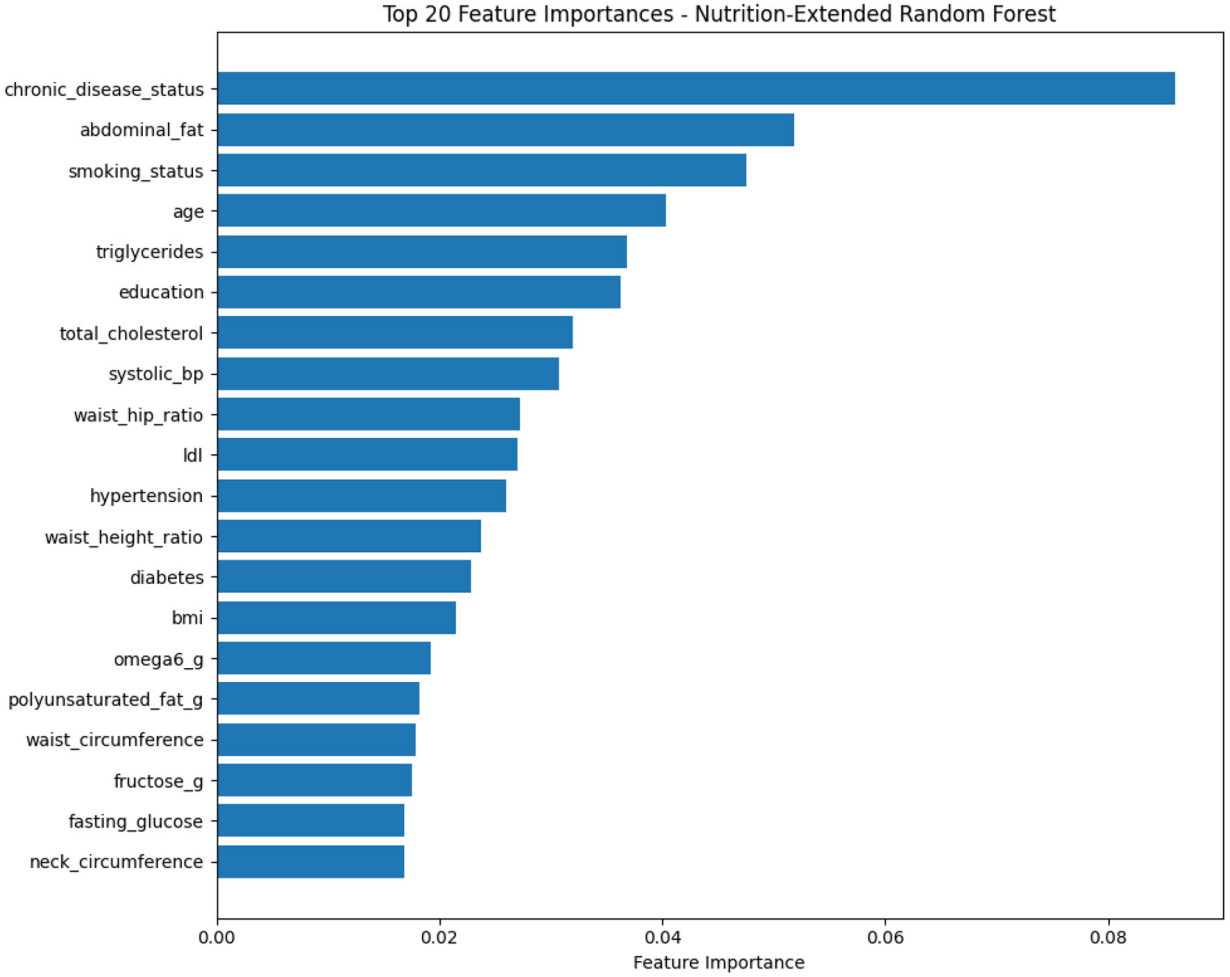
Feature importance plot for the nutrition-extended Random Forest model.

To further investigate the contribution of individual predictors to model performance, feature importance analysis was conducted using the Random Forest classifier. The ranked importance values for the top predictors in the baseline and nutrition-extended models are presented in Tables 9, 10, respectively, while their graphical representations are shown in Figures 8, 9.

**Table 9:** Top feature importance rankings in the baseline Random Forest model.

Rank	Feature	Importance
1	chronic_disease_status	0.091651
2	abdominal_fat	0.069204
3	age	0.053711
4	smoking_status	0.052930
5	education	0.045330
6	triglycerides	0.044684
7	total_cholesterol	0.044276
8	ldl	0.041672
9	systolic_bp	0.036228
10	waist_hip_ratio	0.035713
11	waist_height_ratio	0.034486
12	hypertension	0.030191
13	bmi	0.028369
14	neck_circumference	0.027154
15	fasting_glucose	0.027075
16	waist_circumference	0.026869
17	weight	0.026686
18	hdl	0.026673
19	hip_circumference	0.026538
20	height	0.026131

**Table 10:** Top feature importance rankings in the nutrition-extended Random Forest model.

Rank	Feature	Importance
1	chronic_disease_status	0.086024
2	abdominal_fat	0.051841
3	smoking_status	0.047523
4	age	0.040368
5	triglycerides	0.036817
6	education	0.036234
7	total_cholesterol	0.032013
8	systolic_bp	0.030751
9	waist_hip_ratio	0.027273
10	ldl	0.027057
11	hypertension	0.025947
12	waist_height_ratio	0.023748
13	diabetes	0.022825
14	bmi	0.021512
15	omega6_g	0.019178
16	polyunsaturated_fat_g	0.018177
17	waist_circumference	0.017906
18	fructose_g	0.017524
19	fasting_glucose	0.016870
20	neck_circumference	0.016848

In the baseline Random Forest model (Table 9; Figure 8), chronic_disease_status emerged as the most influential predictor with an importance score of 0.0917, substantially higher than all other variables. This was followed by abdominal_fat (0.0692), age (0.0537), and smoking_status (0.0529). Additional important predictors included education (0.0453), triglycerides (0.0447), total_cholesterol (0.0443), and ldl (0.0417). Anthropometric indicators such as waist_hip_ratio, waist_height_ratio, bmi, and waist_circumference, as well as clinical variables including hypertension and fasting_glucose, also ranked among the top contributors. Overall, the baseline importance distribution indicates that the model primarily relied on a combination of chronic disease status, adiposity measures, age, smoking behavior, and lipid-related biomarkers.

In the nutrition-extended Random Forest model (Table 10; Figure 9), chronic_disease_status remained the most important predictor (0.0860), confirming its dominant role even after the inclusion of dietary variables. The next most important features were abdominal_fat (0.0518), smoking_status (0.0475), and age (0.0404), followed by triglycerides (0.0368) and education (0.0362). Other non-nutritional predictors, including total_cholesterol, systolic_bp, ldl, hypertension, and waist-related ratios, continued to rank highly.

Notably, several nutritional variables entered the top 20 in the nutrition-extended model. These included omega6_g (0.0192), polyunsaturated_fat_g (0.0182), and fructose_g (0.0175). However, these variables were positioned in the lower half of the ranking and exhibited substantially lower importance values compared to the leading predictors. None of the nutritional variables appeared among the top ten features.

A direct comparison between Tables 9 and 10 shows that the inclusion of nutritional variables led to a redistribution of importance scores rather than a change in the dominant predictors. The importance values of key variables such as abdominal_fat (0.0692 → 0.0518), age (0.0537 → 0.0404), and triglycerides (0.0447 → 0.0368) decreased slightly, indicating that part of the predictive contribution was shared with newly introduced dietary variables. However, these changes were relatively small and did not alter the overall ranking structure.

Importantly, the strongest predictors in both models remained non-nutritional variables, particularly those related to existing disease status, adiposity, metabolic health, and lifestyle factors. The dietary variables, although present in the model, contributed only modest and secondary predictive value.

These findings provide a direct explanation for the earlier model performance results. Although nutritional variables increased the dimensionality of the dataset, they did not introduce sufficiently strong or independent predictive signals to improve the performance of the Random Forest model. Instead, the model continued to rely predominantly on traditional cardiovascular risk factors. This explains why the nutrition-extended Random Forest model did not outperform the baseline model, despite the inclusion of additional dietary features.

Overall, the feature importance analysis indicates that clinical, anthropometric, and lifestyle-related variables remain the primary drivers of cardiovascular disease prediction, whereas nutritional variables contribute a limited, model-dependent, and incremental effect within the Random Forest framework.

## 5 Discussion

The present study examined whether detailed nutritional variables improve cardiovascular disease (CVD) prediction when added to a baseline set of demographic, lifestyle, anthropometric, biochemical, and clinical predictors. The results showed that machine learning models were able to achieve moderate discriminatory performance overall, but the contribution of nutritional variables was limited, model-dependent, and incremental rather than consistently beneficial. The strongest overall performance was obtained with the baseline Random Forest model, whereas the inclusion of nutritional variables produced only limited and incremental gains in selected models such as SVM and ANN/MLP and did not improve the best-performing baseline classifier. This suggests that, in the present dataset, conventional cardiovascular risk-related variables already captured most of the dominant predictive signal, while dietary variables contributed only a smaller complementary effect.

One of the more important findings is that, under the labeling convention adopted in this study, specificity values—reflecting the correct identification of individuals with cardiovascular disease—were generally lower than recall values, which reflect the correct identification of individuals without cardiovascular disease. From a clinical screening perspective, this pattern indicates that the models were more effective at recognizing individuals without cardiovascular disease than at detecting those with the condition. This represents an important limitation, since in a disease-screening context, the failure to identify true disease cases may carry greater clinical consequences than the generation of false positives. The relatively lower specificity values therefore suggest that, despite the application of SMOTE to address class imbalance, the models still exhibited a tendency to under-detect the minority (diseased) class. This finding highlights the need for further calibration, threshold optimization, or alternative resampling strategies before such models could be considered for practical screening applications.

Among all algorithms evaluated, Random Forest showed the highest overall performance under the baseline configuration. This result is in line with several previous studies reporting that tree-based ensemble methods perform strongly in cardiovascular prediction tasks. For example, Gupta and Seth (23) found that Random Forest achieved the best performance in their comparative analysis of machine learning and deep learning models, while Noori Mohammad Ali and Muhammed Ahmed (29) also reported Random Forest as the strongest model in hospital-based CVD prediction.

However, the present study differs from many earlier works in an important way: its central aim was not only to identify the best-performing classifier, but to evaluate the incremental value of nutritional variables under otherwise constant analytical conditions. From this perspective, the findings are particularly informative. The fact that the baseline Random Forest model outperformed the nutrition-extended Random Forest model indicates that adding dietary intake variables did not strengthen the most successful classifier. Instead, the added nutritional information appears to have redistributed feature importance without generating enough independent signal to improve overall discrimination. This pattern reinforces that the predictive contribution of nutrition is limited, model-dependent, and incremental, and cannot be assumed to enhance performance automatically.

This interpretation is supported by the feature importance analysis. In both the baseline and nutrition-extended Random Forest models, the leading predictors were overwhelmingly non-nutritional variables, particularly chronic disease status, abdominal fat, age, smoking status, triglycerides, total cholesterol, LDL, and blood pressure-related measures. Although selected dietary variables such as omega-6 intake, polyunsaturated fat intake, and fructose intake entered the top-ranked features in the nutrition-extended model, they remained in the lower half of the ranking and had substantially lower importance values than the strongest clinical and anthropometric predictors. Therefore, the dietary variables were not irrelevant, but their role was limited, model-dependent, and incremental rather than central. In practical terms, the model still relied primarily on traditional cardiovascular risk factors, which helps explain why the Random Forest model did not improve after the inclusion of nutritional variables.

The present findings only partially align with previous nutrition-focused prediction studies. Rigdon and Basu (31) reported that raw nutrition variables, when combined with machine learning, substantially improved cardiovascular mortality risk prediction beyond standard models, with the best performance observed when raw dietary data were used rather than composite diet indices. Likewise, Martin-Morales et al. (23) found that mixed models combining health and nutrition variables outperformed health-only and nutrition-only models, and they highlighted the value of dietary intake in CVD mortality prediction. Compared with those studies, the present results are more conservative: although some models showed limited and incremental improvements after the inclusion of nutrition, this effect was model-dependent and did not enhance the best-performing classifier.

Several methodological differences may help explain this discrepancy. First, some of the prior studies focused on CVD mortality or long-term risk outcomes rather than current disease status. Dietary variables may have a stronger relationship with long-term prognosis than with cross-sectional disease classification. Second, studies such as Rigdon and Basu (31) and Martin-Morales et al. (18) used NHANES-based designs with broader food- or nutrient-level representations, whereas the present study was based on a city-centered adult sample with a different demographic and nutritional structure. Third, the quality and granularity of dietary measurement may differ substantially across studies. The current dataset relied on a 24-hour dietary recall, which is practical and widely used but is also sensitive to recall bias, short-term intake fluctuation, and under- or over-reporting. These sources of error may weaken the independent predictive contribution of nutrition variables, especially when strong biochemical and anthropometric predictors are already present. Rigdon and Basu (31) themselves noted both the sparsity and complexity of nutrition data and emphasized that machine learning may be particularly useful when attempting to exploit this kind of structure.

The current results are also informative when viewed alongside population-based nutritional prediction studies. Morgenstern et al. (17) developed prediction models using detailed nutrition variables together with socio-demographic and behavioral factors and obtained competitive predictive performance, while identifying supplement use, caffeine, and alcohol as important dietary predictors. Their work showed that nutrition-based models can be informative even in the absence of clinical and laboratory variables. In contrast, the present study included a much richer baseline clinical and anthropometric profile, and under those conditions the relative contribution of nutrition became smaller. This distinction is important: when strong conventional predictors are already available, the marginal utility of dietary variables may decrease. Thus, the value of nutrition may depend not only on the disease process itself, but also on the strength of the baseline feature space against which it is evaluated.

Another important observation in the present study is that the effect of nutritional variables was model-dependent. SVM and ANN/MLP showed limited and incremental improvements after the addition of dietary variables, whereas Random Forest, Decision Tree, and KNN did not consistently benefit. This pattern suggests that nutrition-related information may be embedded in relationships that some classifiers can exploit more effectively than others. Margin-based and neural models may benefit from subtle and distributed non-linear patterns, whereas tree-based methods may be more sensitive to the introduction of weak or noisy features that dilute stronger predictors. This interpretation is consistent with the broader literature showing that model architecture matters substantially in CVD prediction. For example, Bhatt et al. (34) found that MLP performed best in their clustering-based pipeline, whereas Sadr et al. (30) reported strong performance from hybrid and ensemble strategies combining machine learning and deep learning models. The current findings therefore reinforce the idea that the predictive value of a feature group cannot be evaluated independently of the model used to learn from it.

The current results also intersect with studies emphasizing preprocessing, resampling, and feature selection. Alfebi and Anasanti (26) showed that imputation, imbalance resampling, and feature selection can substantially improve cardiovascular prediction in NHANES data, while Dritsas and Trigka (29) reported strong performance after SMOTE and stacking-based modeling. In the present study, class imbalance was addressed using SMOTE, and this likely contributed to the relatively strong recall values observed across models. However, the persistence of low specificity indicates that balancing the training data alone does not fully resolve the broader classification difficulty. This suggests that, beyond resampling, further gains may require more targeted feature engineering, calibration strategies, or clinically informed threshold optimization.

From a clinical interpretation standpoint, the importance rankings in this study are reassuring because they align closely with established cardiovascular knowledge. Age, smoking status, adiposity measures, lipid abnormalities, and blood pressure-related variables were repeatedly among the strongest predictors. This concordance improves the interpretability and face validity of the models. At the same time, it is important not to overstate this point: agreement with known risk factors is necessary, but it does not by itself prove superiority over conventional statistical modeling. The real added value of machine learning lies in its capacity to model non-linear patterns, interactions, and high-dimensional structures that may not be captured adequately through simpler approaches. In the present study, that advantage appears to be present to some extent, but not strong enough to turn nutritional variables into universally high-impact predictors.

Taken together, the results suggest that nutritional variables should not be viewed as irrelevant, but neither should they be assumed to consistently enhance CVD prediction. Their role is limited, model-dependent, and incremental, depending on the outcome being predicted, the quality of dietary measurement, the richness of the baseline predictors, and the type of algorithm employed. In this study, conventional clinical, anthropometric, and lifestyle-related variables remained the primary drivers of prediction, while dietary variables offered only a limited and incremental complementary layer of information. This is, in itself, an important contribution: it shows that the effect of nutrition must be tested systematically rather than assumed, and that a larger feature set does not necessarily translate into better model performance.

Finally, the present study contributes methodologically by using a controlled comparative design in which the analytical pipeline was held constant and only the feature configuration was changed. This enables a clearer interpretation of what the dietary variables added—and, equally importantly, what they did not add—to cardiovascular disease prediction. Future studies using repeated dietary recalls, food frequency questionnaires, longitudinal outcomes, external validation cohorts, and interpretable post-hoc methods such as SHAP may provide a more refined understanding of when and how nutritional variables meaningfully improve machine learning-based cardiovascular risk assessment.

In addition, the public release of this dataset is expected to contribute to the research community by providing a rare and comprehensive data source that integrates nutritional, clinical, and biochemical variables. Such datasets remain limited in the literature, particularly in the context of machine learning applications for cardiovascular risk prediction. Therefore, making this dataset openly accessible may facilitate reproducibility, enable methodological comparisons, and support the development of more advanced predictiv models.

## 6 Limitations and future perspectives

Despite the strengths of this study, several limitations should be acknowledged when interpreting the findings.

First, the study is based on a cross-sectional dataset, which limits the ability to establish causal relationships between predictors and cardiovascular disease. The models developed in this study are designed for classification rather than causal inference, and therefore the identified associations should be interpreted as predictive rather than explanatory.

Second, the dietary intake data were collected using a single 24-h dietary recall, which is subject to recall bias, reporting inaccuracies, and day-to-day variability in food consumption. This approach may not adequately reflect habitual dietary patterns, which are more relevant for long-term cardiovascular risk. As a result, the predictive contribution of nutritional variables may be underestimated or, in some cases, misrepresented. Future studies should consider using repeated dietary recalls or combining them with food frequency questionnaires (FFQ) to obtain more reliable estimates of usual intake.

Third, the dataset exhibited class imbalance, with a relatively smaller proportion of individuals classified as having cardiovascular disease. Although this issue was addressed using the SMOTE technique, synthetic resampling methods may introduce artificial patterns into the data and do not fully replace the need for naturally balanced datasets. Future research using larger and more balanced cohorts would help validate the robustness of the findings.

An additional consideration concerns the class labeling convention used in this study. Because the positive class (label 1) was defined as the absence of cardiovascular disease (the majority class), the reported recall values primarily reflect the model's ability to correctly classify non-diseased individuals, whereas specificity reflects the model's ability to detect actual cardiovascular disease cases. Although this convention is internally consistent and does not affect threshold-independent metrics such as ROC-AUC, future work adopting the alternative convention (positive class = diseased) may provide complementary insights, particularly for direct comparison with clinical screening literature.

Another limitation concerns the lack of external validation. The models were trained and evaluated using a single dataset derived from a specific population. Therefore, the generalizability of the findings to other populations, age groups, or geographic regions remains uncertain. External validation using independent cohorts is essential to confirm the reliability and clinical applicability of the proposed models.

In addition, although multiple machine learning algorithms were compared, the study did not include advanced ensemble methods (e.g., stacking, boosting) or more complex deep learning architectures beyond standard ANN/MLP models. Furthermore, model interpretability techniques, such as SHAP or LIME, were not applied. The absence of these explainability tools limits the ability to fully understand the direction and magnitude of individual predictor effects at the patient level, which is an important requirement for clinical decision-support systems.

Finally, although the study aimed to evaluate the contribution of nutritional variables, other potentially relevant factors—such as physical activity, genetic predisposition, medication use, and socioeconomic context—were not comprehensively incorporated into the models. These factors may interact with dietary patterns and influence cardiovascular risk, and their omission may limit the completeness of the predictive framework.

Future research should focus on addressing these limitations and further exploring the role of nutritional variables in cardiovascular disease prediction. In particular, longitudinal study designs would enable the investigation of temporal relationships between diet and disease development, providing a more robust basis for risk prediction.

The integration of more precise and repeated dietary assessment methods, including multi-day recalls and FFQ-based approaches, may improve the quality of nutritional data and reveal stronger predictive relationships. In addition, combining dietary data with objective biomarkers of nutritional status could enhance model performance and reduce measuremen bias.

From a methodological perspective, future studies should explore the use of advanced machine learning techniques, including ensemble learning (e.g., stacking, XGBoost, LightGBM) and more sophisticated deep learning architectures. Incorporating explainable artificial intelligence (XAI) methods, such as SHAP, would also improve model transparency and facilitate clinical interpretation.

Moreover, validating the developed models on external and more diverse populations is essential to ensure generalizability. Expanding the dataset to include individuals from different age groups, socioeconomic backgrounds, and geographic regions would strengthen the robustness of the findings.

Finally, future work should aim to develop integrated, clinically applicable decision-support systems that combine nutritional, clinical, and lifestyle data to support personalized risk assessment and early intervention strategies in cardiovascular health.

## 7 Conclusions

This study evaluated the contribution of detailed nutritional variables to cardiovascular disease prediction using multiple machine learning algorithms under two feature configurations: a baseline dataset and a nutrition-extended dataset. The findings showed that the predictive effect of nutritional variables was limited, model-dependent, and incremental. Although limited and incremental improvements were observed in selected models, particularly SVM and ANN/MLP, the best overall performance was achieved by the baseline Random Forest model. This indicates that conventional predictors—especially chronic disease status, adiposity-related measures, smoking behavior, blood pressure, and lipid-related biomarkers—remain the dominant determinants of cardiovascular disease prediction in this dataset.

Feature importance analysis further supported this conclusion by showing that nutritional variables contributed only a limited, model-dependent, and incremental predictive value compared with established clinical, anthropometric, and lifestyle-related factors. Therefore, the inclusion of dietary variables did not consistently improve predictive performance across all classifiers and should not be assumed to provide universal benefit in machine learning-based cardiovascular disease prediction.

Overall, the present study demonstrates that nutritional variables offer limited, model-dependent, and incremental complementary information, the usefulness of which depends on the modeling approach and the strength of the baseline predictors already available. These findings highlight the importance of evaluating feature groups systematically rather than assuming that a larger feature set will necessarily lead to better predictive performance. Future studies using larger cohorts, repeated dietary assessments, and external validation datasets may help clarify the long-term and context-specific predictive value of nutritional information in cardiovascular disease modeling.

## Data Availability

The raw data supporting the conclusions of this article will be made available by the authors, without undue reservation.
