# Supplementary material for: Do nutritional variables improve cardiovascular disease prediction? A comparative machine learning analysis
Source: Front Nutr. 2026 Jul 8;13:1808942. doi: 10.3389/fnut.2026.1808942 (PMC13390704; doi:10.3389/fnut.2026.1808942)

**Supplementary Table 1. Generative AI Input Prompt and Output Details for Figure 1**

- **Name of generative AI technology:** SciSpace Generate Diagram
- **Source:** SciSpace
- **Version:** Not specified/displayed by the platform
- **Model:** Not specified/displayed by the platform

**1. Input Prompt Used to Generate Figure 1:**

‘Generate a scientific diagram in a clean infographic style. Use horizontal or vertical panels if needed to show steps in sequence.

Create a high-quality scientific workflow diagram for a machine learning study on cardiovascular disease prediction. The diagram should be clean, minimal, and suitable for publication in a Q1 journal.

The workflow should include the following steps in order:

- Raw dataset (n = 1,359, 62 variables after removing ID)
- Data preprocessing:
  - Missing value handling (cigarettesperday filled with 0)
  - Outlier inspection (retained, not removed)
  - Train-test split (80/20, stratified)
  - Feature scaling (Min-Max normalization, Equation 1)
- Feature grouping into two parallel datasets:
  - Baseline dataset (demographic, lifestyle, anthropometric, biochemical, clinical)
  - Nutrition-extended dataset (baseline + dietary variables)
- Apply SMOTE (only on training data, separately for each dataset)
- Model training:
  - Logistic Regression
  - Random Forest
  - SVM
  - KNN
  - Decision Tree
  - ANN
- Model evaluation on test set:
  - Accuracy, Precision, Recall, F1-score, ROC-AUC, Specificity
- Final comparison:
  - Performance difference between baseline and nutrition-extended datasets

The diagram should clearly show two parallel pipelines after feature grouping (baseline vs nutrition-extended) and highlight the comparison step at the end.

Use a modern academic style:

- white background
- soft colors (blue/green/gray)
- rectangular blocks with arrows
- minimal icons (optional)
- no clutter
- readable text

Make it suitable for inclusion as a figure in a scientific article.’

**2. Generated Output:**


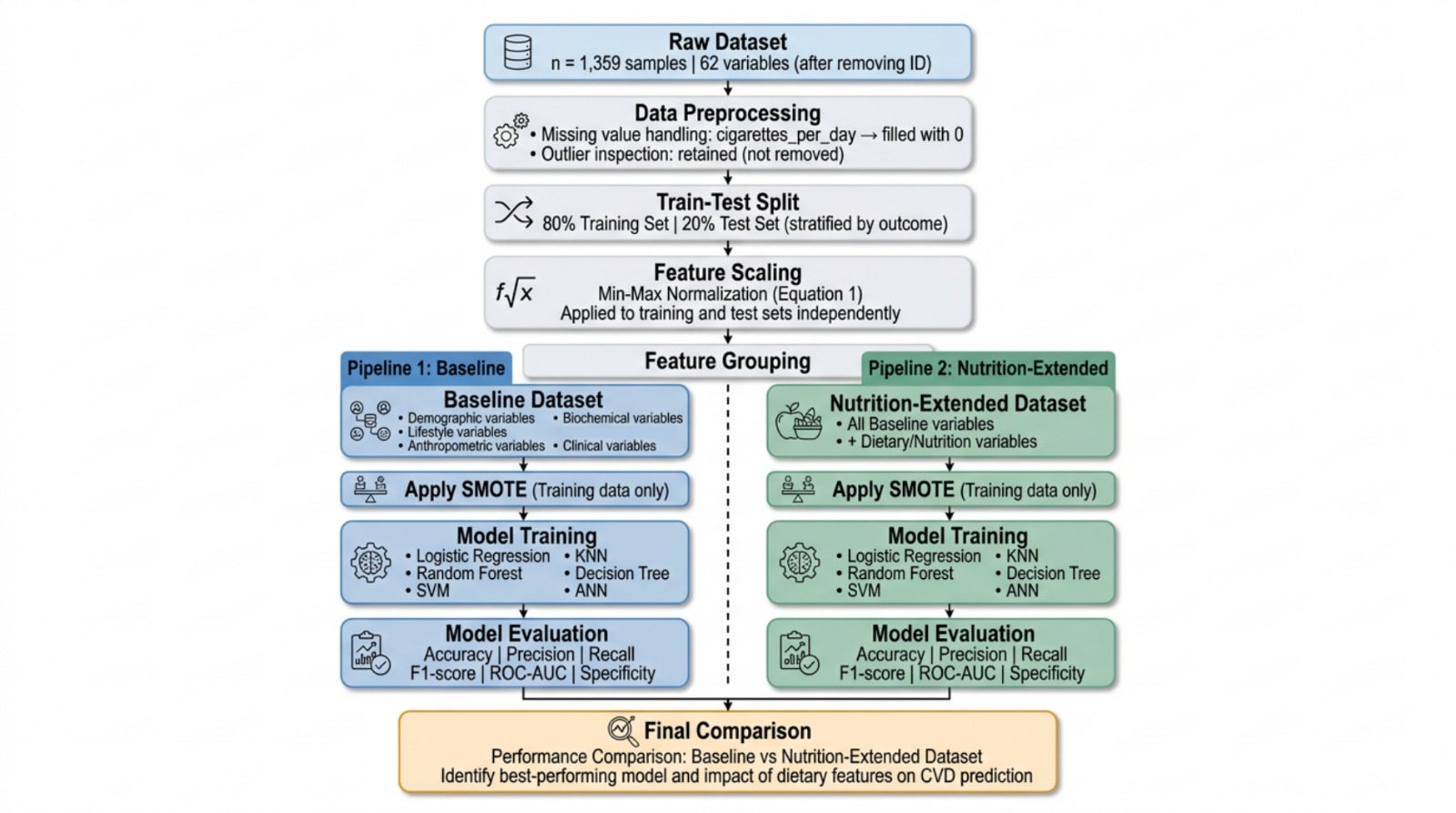

Supplement: Supplementary file 1 [file Table_1.docx]
